# Diarylethene‐Based Photoswitchable Inhibitors of Serine Proteases

**DOI:** 10.1002/anie.202108847

**Published:** 2021-08-25

**Authors:** Oleg Babii, Sergii Afonin, Christian Diel, Marcel Huhn, Jennifer Dommermuth, Tim Schober, Serhii Koniev, Andrii Hrebonkin, Alexander Nesterov‐Mueller, Igor V. Komarov, Anne S. Ulrich

**Affiliations:** ^1^ Institute of Biological Interfaces (IBG-2) Karlsruhe Institute of Technology (KIT) POB 3640 76021 Karlsruhe Germany; ^2^ Institute of Microstructure Technology (IMT) KIT Hermann-von-Helmholtz-Platz 1 76344 Eggenstein-Leopoldshafen Germany; ^3^ Institute of Organic Chemistry (IOC) KIT Fritz-Haber-Weg 6 76131 Karlsruhe Germany; ^4^ Taras Shevchenko National University of Kyiv vul. Volodymyrska 60 1601 Kyiv Ukraine; ^5^ Lumobiotics GmbH Auer Straße 2 76227 Karlsruhe Germany

**Keywords:** bicyclic peptide, diarylethene photoswitch, hydrogel photoregulation, serine protease inhibitors, trypsin

## Abstract

A bicyclic peptide scaffold was chemically adapted to generate diarylethene‐based photoswitchable inhibitors of serine protease Bos taurus trypsin 1 (T1). Starting from a prototype molecule—sunflower trypsin inhibitor‐1 (**SFTI‐1**)—we obtained light‐controllable inhibitors of T1 with K_i_ in the low nanomolar range, whose activity could be modulated over 20‐fold by irradiation. The inhibitory potency as well as resistance to proteolytic degradation were systematically studied on a series of 17 **SFTI‐1** analogues. The hydrogen bond network that stabilizes the structure of inhibitors and possibly the enzyme–inhibitor binding dynamics were affected by isomerization of the photoswitch. The feasibility of manipulating enzyme activity in time and space was demonstrated by controlled digestion of gelatin‐based hydrogel and an antimicrobial peptide BP100‐RW. Finally, our design principles of diarylethene photoswitches are shown to apply also for the development of other serine protease inhibitors.

## Graphical abstract

Bicyclic diarylethene‐containing analogues of the sunflower trypsin inhibitor‐1 (**SFTI‐1**) can modulate activity of serine proteases in the nanomolar–micromolar range in highly efficient photocontrollable manner.

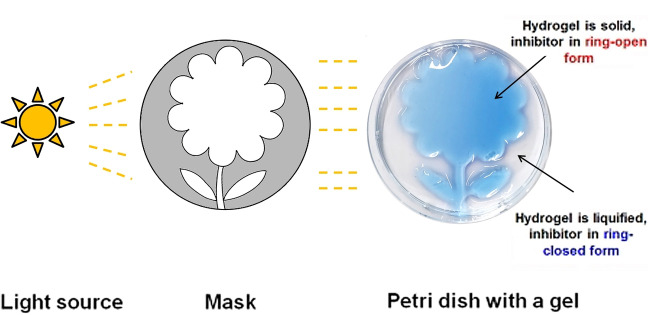

The use of light to control functions of biomacromolecules has become an active field of research over the last decades. Reversibly photoisomerizable (i.e. photoswitchable) compounds have already demonstrated great promise in medicinal chemistry^[1]^ and materials science.^[2]^ Particular attention has been devoted to control the activity of enzymes by light,^[3]^ as enzymes are important drug targets^[4a]^ and often are components of “smart” soft materials.[^4b^, ^4c^] Most of the known photocontrollable enzyme inhibitors have been designed using two general strategies, illustrated in Figure 1. One (Figure 1 A) is based on the incorporation of a photoisomerizable fragment (photoswitch) into a known inhibitor as part of the enzyme‐binding moiety. This strategy is well‐suited for the design of small‐molecule constructs; many photocontrollable, mostly azoarene‐derived inhibitors[^5a^, ^5b^, ^5c^, ^5d^] of this type have been reported, although other types of photoswitches were also used.[^5e^, ^5f^, ^5g^, ^5h^] Another successful approach consists of designing so‐called “two‐pronged” inhibitors, in which the photoswitching unit connects two target‐binding moieties (Figure 1 B). Efficient diarylethene (DAE)^[6]^ and azobenzene‐modified^[7]^ enzyme modulators with two distinct binding sites have been prepared using this second approach. Here, we introduce yet another design—grafting the target‐binding and the photoswitching moieties onto opposing loops of a macro‐bicyclic scaffold (Figure 1 C). In this case, the constructs have the binding and photoregulating units joined through a bridge in the bicycle.

**Figure 1:**
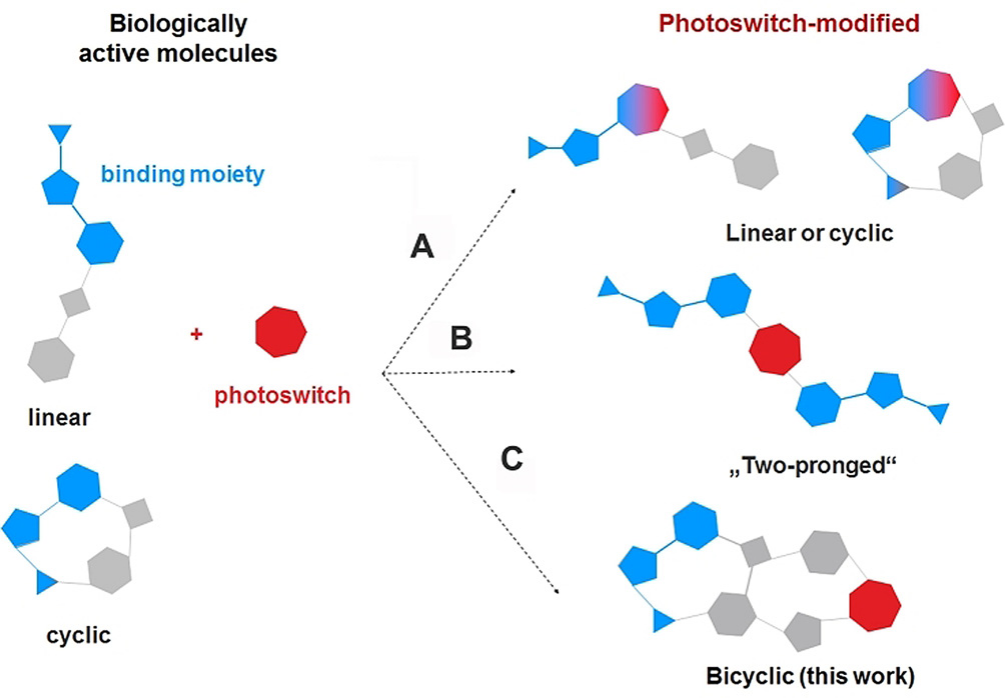
General design strategies of photoswitchable enzyme inhibitors. The moieties directly binding to enzymes are schematically shown in blue, the photoswitch is shown in red.

We were inspired by success in using bicyclic scaffolds for design of non‐photocontrollable multifunctional peptides. For example, Pei et al. reported recently on bicyclic cell‐permeable PIN‐1 inhibitors,^[8]^ in which the enzyme‐binding and cell‐penetrating units were joined through a bicyclic peptide core. Although the photoswitch moiety is distant to the binding unit in our constructs, we demonstrate here that both units can “communicate” through the bicyclic scaffold, resulting in efficient photomodulation.

With the suggested strategy, we designed and optimized DAE‐based peptidic inhibitors of chymotrypsin‐like (S1) serine proteases, the largest family of all peptidases.^[9]^ Serine proteases have a broad biological significance and immense potential as drug targets, as their malfunction is linked to numerous pathologies.^[10]^ These enzymes have been in the focus of intensive research directed towards the discovery of new inhibitors,^[11]^ including photocontrollable ones.^[12]^ The latter were based exclusively on azobenzenes as the photoswitching moiety; no DAE‐derived inhibitors have been reported to date for this class of enzymes.

*Bos taurus* trypsin 1 (T1) represents the S1 family by its catalytic mechanism, inhibitor susceptibility profile and substrate specificity, and was thus selected as the model target.^[9]^ As the reference compound and as the starting template, we used the sunflower trypsin inhibitor‐1 (**SFTI‐1**). It is bicyclic, well‐studied and is one of the strongest natural inhibitors of trypsin‐like endopeptidases (*K*_i_ 0.1–3.4 nm for T1).^[13]^ The **SFTI‐1** molecule has two antiparallel β‐strands stabilized by a disulfide bridge (Figure 2).^[14]^

**Figure 2:**
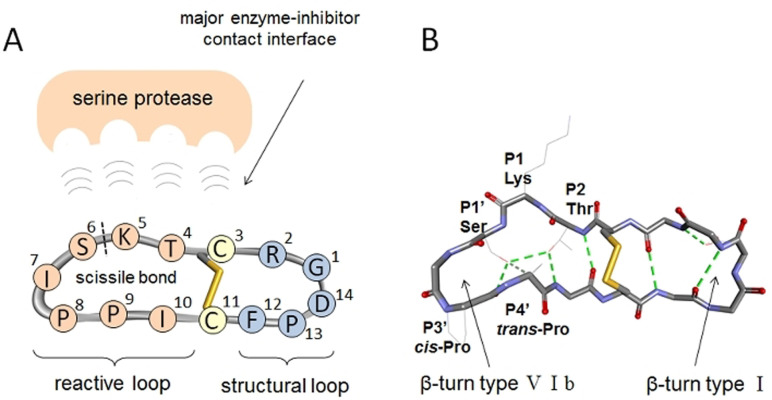
Sequence, nomenclature and structure of **SFTI‐1**. A) Schematic representation of the **SFTI‐1** peptide and its structural/functional parts. B) Molecular structure and hydrogen bonding network (PDB: 1JBL).^[14a]^

When designing the analogues of the **SFTI‐1**, we relied on the known mechanism of inhibition and the relationship between structure and activity of **SFTI‐1**.[^9^, ^15^] We also considered the changes in molecular dynamics that the diarylethene moiety causes upon photoisomerization.[^5e^, ^16^] **SFTI‐1** inhibits trypsin by the Laskovski mechanism,^[17]^ that is, it binds tightly to the protease (Figure 2 A) but resists hydrolysis, thereby blocking the enzyme. The resistance to hydrolysis has been attributed[^13a^, ^14a^, ^18^] to a rigid bicyclic structure and a tight network of hydrogen bonds (Figure 2 B).

Modifications of the reactive loop are known to drastically deteriorate the inhibitory activity, while the structural loop can be modified without substantial activity drop.[^13a^, ^19^] Therefore, we incorporated the photoswitch within the structural loop of **SFTI‐1**, using building block **1** (Figure 3) developed previously by us.^[20]^ We aimed at the compounds in which the ring‐open DAE fragment would cause minor changes in the enzyme inhibition, while the ring‐closed DAE photoform would minimize this activity.

**Figure 3:**
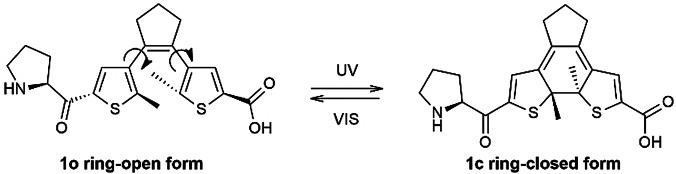
DAE‐containing amino acid used to prepare the **SFTI‐1** analogues, and nomenclature of the photoisomers.

The first series of DAE‐containing **SFTI‐1** analogues (**S1n**, **S2**–**S4**, Table 1) was prepared to explore the impact of the structural loop size upon the inhibitory activity and photoswitching efficiency, and to check the role of the bridge on the functionality of the entire construct. As can be seen from the data in Table 1, compound **S1n** proved to be a strong T1 inhibitor, comparable to **SFTI‐1**. However, this peptide could not be converted to the ring‐closed form, presumably because of severe constraints in the structural loop due to its small size.^[21]^ The analogue **S2**, lacking the bridge, was well photoisomerizable. (See the data about the photostationary state under UV irradiation for all the compounds studied here in the SI. Conversion from ring‐closed forms to the ring‐open forms under visible light irradiation (at 570 nm) was in all cases practically quantitative.) However, we observed a significant drop in its activity compared to the native inhibitor. The smaller bridge‐free analogue **S3** also showed a reduced activity. Besides, it was obtained with a very low yield at the cyclization step. The smallest bridge‐free analogue **S4** (ring‐open), although being a potent inhibitor, also did not photoisomerize into the ring‐closed form. These results from the first series suggest that incorporation of the DAE into the structural loop of **SFTI‐1** may indeed result in potent T1 inhibitors. However, more conformational freedom should be provided in the structural loop to enable effective photoisomerization, and the bridge should be kept in place.

**Table 1:** T1 inhibitors studied in this work.^[a]^

Name	Sequence	*K*_i_ [nM] (open)	*K*_i_ [nM] (closed)	*K*_i_ (closed) /*K*_i_ (open)	*k*_H_ [M^−1^ s^−1^] (open)	*k*_H_ [M^−1^ s^−1^] (closed)	*k*_H_ (closed) /*k*_H_ (open)
*Reference compounds*							
**SFTI‐1**	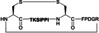	3.4±0.2		–	2.0±0.1×10^−5^		–
**SFTI‐1i**	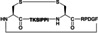	12.5±0.8		–	2.0±0.2×10^−5^		–
					
*First series of inhibitors*							
**S1n**	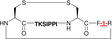	4.9±0.3	–	–	7.8±0.2×10^−5^	–	–
**S2**	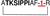	203±6	659±33	3.2	–	–	–
**S3**	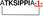	101±9	–	–	–	–	–
**S4**	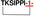	22±3	–	–	–	–	–
							
*Second series*							
**S1i**	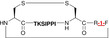	13.4±1.4	–	–	8.3±0.3×10^−5^	–	–
**S5n**	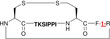	95±6	231±14	2.4	2.5±0.1×10^−3^	3.4±0.5×10^−2^	13.6
**S5i**	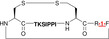	102±14	182±16	1.8	2.9±0.1×10^−3^	5.0±0.8×10^−3^	1.7
**S6n**	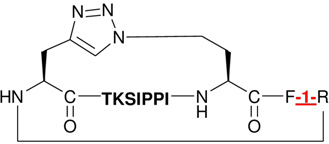	135±8	1120±109	8.3	6.7±0.9×10^−3^	2.5±0.8×10^−2^	3.7
**S6i**	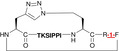	441±43	7700±600	17.5	1.9±0.1×10^−2^	1.1±0.2×10^−1^	5.8
**S7n**	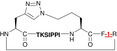	63.4±4.4	2200±214	34.7	2.0±0.2×10^−3^	6.1±1.2×10^−2^	30.5
**S7i**	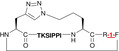	263±30	7220±360	27.5	7.5±0.2×10^−3^	5.5±1×10^−1^	73
							
*Third series*							
**S8n**	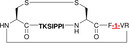	5.2±0.4	125±8	24	1.3±0.7×10^−4^	5.3±1.4×10^−4^	4.1
**S9n**	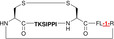	8.3±0.6	55±3.8	6.6	1.5±0.3×10^−4^	1.3±0.1×10^−3^	8.7
**S10n**	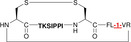	17.5±0.7	181±7	10.3	2.3±0.2×10^−4^	3.6±0.3×10^−3^	15.7
**S10i**	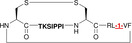	5.1±0.4	44.8±2.2	8.8	5.3±0.4×10^−5^	2.2±0.3×10^−4^	4.2
**S11i**	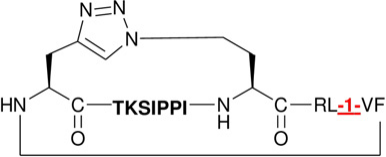	1720±130	5370±420	3.1	2.8±0.3×10^−2^	9.1±1×10^−2^	3.3
**S12i**	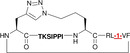	2440±215	12 400±900	5.0	1.4±0.3×10^−2^	4.2±0.4×10^−2^	3.0

[a] T1 inhibitory activities (*K*_i_), T1‐induced hydrolysis rate constants (*k*_H_), and their ratio for the ring‐closed to the ring‐open isomers. The residue of **1** is highlighted by red color and underlined.

We hypothesized that an extension of the bridge might provide the required freedom. Replacement of the disulfide bond in **SFTI‐1** with surrogates has been described in the literature.^[22]^ The majority of such substitutions were functionally tolerated, providing a significant increase in the redox‐stability. Hence, we designed the second series of analogues with elongated bridges (**S5n**–**S7n**, Table 1).

The proteolytic stability of the peptide inhibitors is the second major factor, besides *K*_i_, that is of importance for any practical utility.[^19d^, ^23^] The hydrolysis rate does not strictly correlate with *K*_i_ but depends on the activation barrier of the hydrolysis reaction.^[24]^ It is known that T1 may cleave **SFTI‐1** at Arg2 in the structural loop. Since neither Arg2 nor Phe12 have been reported as critical in binding to trypsin,^[19a]^ we supplemented the second series of **SFTI‐1** analogues by “inverted” compounds. Here, Arg2 and Phe12 were swapped in order to evaluate the effect of Arg2 on the proteolytic stability (**S1i**, **S5i**–**S7i**, Table 1). As anticipated, all analogues with elongated bridges (**S5n**–**S7n**, **S5i**–**S7i**) were photoisomerizable. Generally, upon extending the bridge, the potency and stability of the peptides decreased. At the same time, we observed a systematic increase in the activity difference between the ring‐open and ring‐closed forms. We also found that the Arg2/Phe12 inversion improved the proteolytic stability of some analogues.

In the third series of photoswitchable **SFTI‐1** analogues, we explored the extension of the peptide cycles. One or two additional aliphatic residues (Val and Leu) were introduced at the flanks of the photoswitch (**S8n**–**S10n**, **S10i**, Table 1). Two triazole‐linked analogues (**S11i**, **S12i**) bearing elongated bridges were also prepared. This design yielded the best photoswitchable analogues, which not only retained a high inhibitory potency in the low nanomolar range, but also showed efficient photoswitching of their activity. Compound **S8n**, which is the best performing molecule in terms of inhibitory potency and photoswitching efficiency of the inhibiting activity, was found to change its *K*_i_ from 5.2 to 125 nm upon photoisomerization, that is, by a factor of 24. The hydrolysis rates for the disulfide‐bearing inhibitors of this series were close to that for native **SFTI‐1**. The triazole‐bridged **S11i** and **S12i**, however, showed a drastic loss of activity as well as proteolytic stability.

Structural preorganization—resulting in a low entropic penalty upon enzyme binding—was postulated to be the key factor determining the high potency of **SFTI‐1**.^[13a]^ To understand the mechanism by which the DAE photoswitch in our compounds modulates the *K*_i_ and hydrolysis rate, we measured hydrogen/deuterium exchange rates for the compounds in D_2_O using MALDI mass spectrometry. From these data, the number of hydrogen bonds could be quantified and compared with the values obtained for *K*_i_ and *k*_H_.^[25]^ Figure 4 illustrates the relationship between conformational stability in terms of the number of protons in slow exchange, proteolytic stability, and the potency of each inhibitor/photoisomeric state. The prototype **SFTI‐1** and its “inverted” mutant **SFTI‐1i** possessed 7.0 slow‐exchanging protons (*t*_1/2_>40 s). The best‐performing analogues **S8n**, **S9n**, **S10n**, and **S10i** had a comparable number of slow‐exchanging protons in their ring‐open forms (5.5 to 6.5), but far less upon photoswitching, which correlated well with their *K*_i_ and *k*_H_. This correlation provides evidence that the ring‐open DAE facilitates preorganization of the reactive loop through hydrogen bonds, which stabilize the conformation and enhance the potency.

**Figure 4:**
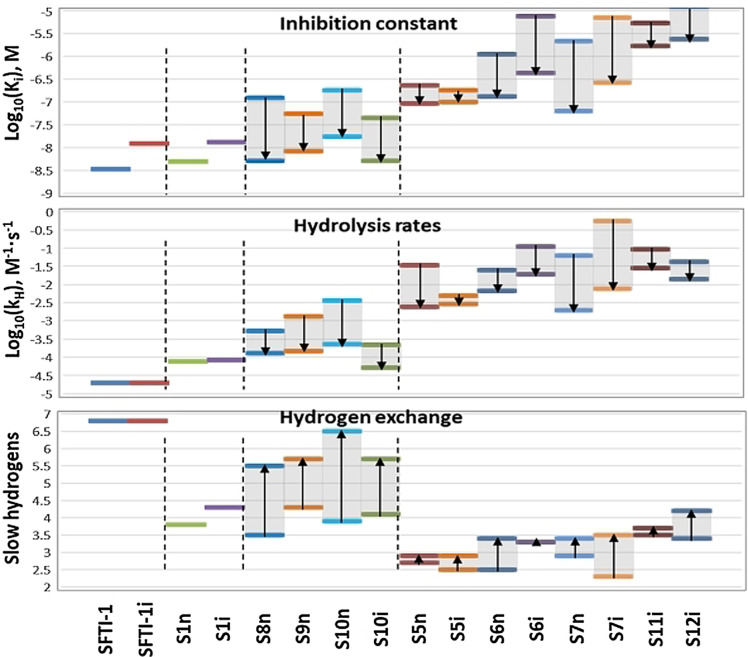
Relationship between T1 inhibitory activity, proteolytic stability, and the number of slow‐exchanging protons in the photoswitchable **SFTI‐1** analogues. The ring‐closed to ring‐open changes are indicated by arrows.

The structure–activity relationship of the entire library is not as straightforward, however. Compound **S6i**, for example, has approximately the same number of exchanging protons for its two photoforms (approx. 3.4), yet its *K*_i_ and *k*_H_ changed significantly upon photoisomerization (Figure 4). Although an influence of the DAE moiety on the structural preorganization of the **S6i** molecule cannot be excluded, it appears more probable that the DAE photoswitching modulates the dynamics of the enzyme–inhibitor complex. The DAE is expected to significantly influence the dynamics, because this photoswitch possesses a very different flexibility in its two photoforms. The more flexible ring‐open peptides can thus have more favorable conformational and vibrational entropy changes upon binding to the target protein than the corresponding ring‐closed photoforms.^[16]^

**SFTI‐1** serves as a lead in developing inhibitors of other serine proteases.[^22c^, ^22e^, ^23b^, ^26^] To demonstrate that our photoswitchable T1 inhibitors can be modified to inhibit other members of the chymotrypsin‐like family, we synthesized **S5F**, an analogue of **S10n** in which Lys5 at the P1 position was changed to Phe. Such modification in the native **SFTI‐1** had been reported to result in a potent inhibitor of α‐chymotrypsin. A non‐photoswitchable mutant **SFTI‐5F** was taken as a control, whose *K*_i_ value was measured to be 2.8 nm. As expected, **S5F** inhibited α‐chymotrypsin (from bovine pancreas Type II) very well. The *K*_i_ values of the two photoisomers differed by 21‐fold (5.8 nm for the ring‐open form, 122 nm for the ring‐closed form), proving that the present scaffold can be adapted to inhibit other serine proteases in the same photocontrollable manner. We also wondered how selective our inhibitors were and characterized **S5F** against trypsin and its prototype **S10 n** (one of the best‐performing trypsin inhibitors) against α‐chymotrypsin. As expected, only weak inhibition with IC_50_ values at 50–100 μm (>3 orders of magnitude lower compared to their parent proteases' inhibition) was observed in each case, proving sufficient enzyme selectivity of our compounds.

Finally, photostability in repeated cycles of reversible photoisomerization was studied on three selected compounds (linear Ac‐Ala‐**1**‐Ala‐NH_2_, peptide **S2** lacking the bridge, and **S10i**). All three compounds demonstrated moderate photofatigue resistance in these tests (see the SI) degrading less than 30 % in 15 cycles. We consider this acceptable for most biomedical applications, where only one or a few cycles of the photoconversion are usually needed.[^1^, ^2^, ^3^]

To demonstrate the manipulation of the enzyme activity in space and time by light, we set up two experiments. The first one was based on digestion of a gelatin‐based hydrogel with trypsin. A water solution of gelatin (20 mg mL^−1^) was mixed with **S10n** (ring‐closed form) and trypsin (10 μm and 100 nm final concentrations, respectively). Bromophenol blue was added for better visualization of the gel, which was formed in a Petri dish at 0 °C in the dark. The light (approx. 10 mW cm^−2^) was then applied for 3 min to the gel trough a mask to convert **S10n** to the ring‐open form, an active trypsin inhibitor. Incubation of the Petri dish at 20 °C after the irradiation resulted in liquefying of the gel only in places where the light did not reach the mixture. The liquid was removed, leaving the intact areas where trypsin was inhibited by the photoactivated **S10n** (Figure 5; an image of the whole Petri dish after the experiment can be seen in the Table of Contents picture).

**Figure 5:**
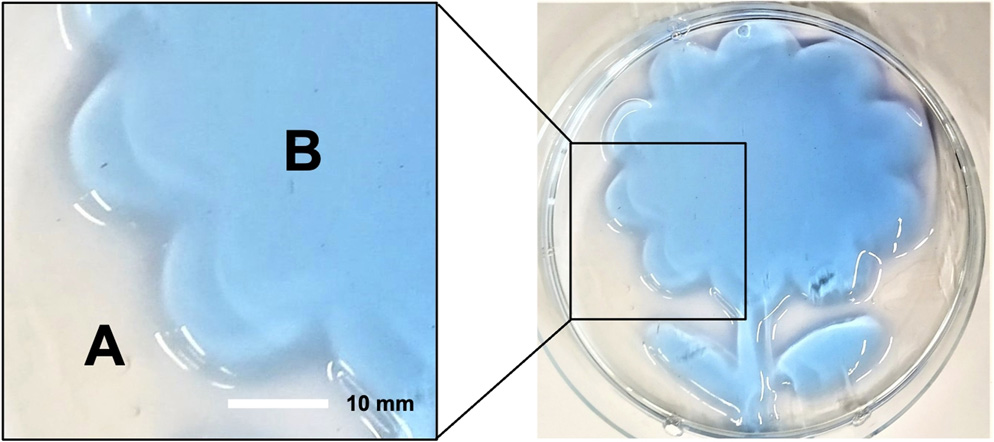
Photocontrolled trypsin digestion of gelatin‐based hydrogel in a Petri dish (right); a magnified part of it is shown in the insert (left): A) the area where **S10n** (ring‐closed) was not irradiated, the gel was digested and removed by a filter paper; B) the area where the ring‐closed **S10n** was converted to the ring‐open photoform and inhibited trypsin. The hydrogel was stained by bromophenol blue.

An analogous experiment was designed to show the photoregulation of BP100‐RW peptide activity by digestion with trypsin. BP100‐RW (sequence RRLFRRILRWL‐NH_2_) is known as a potent antimicrobial peptide (AMP).^[27]^ Growth of *E. coli* DSM 498 was monitored in a media containing the peptide (64 μg mL^−1^, twice as high as the minimal inhibitory concentration), trypsin (3 nm), and **S10n** ring‐closed (300 nm). Irradiation of the mixture prevented the bacterial growth, while the bacteria grew almost as fast as without the BP100‐RW in a control experiment in dark (Figure S9).

In summary, we have evaluated a strategy for the design of efficiently photocontrollable bicyclic peptide‐based enzyme inhibitors. Our compounds possess the enzyme‐binding fragment in one cyclic unit, and the photoswitchable fragment in the opposite cyclic unit of the macro‐bicycle. We used the diarylethene photoswitch for effective regulation of inhibiting activity of serine proteases, which has never been used before for this class of enzymes and demonstrated the utility of the obtained enzyme inhibitors for photoregulation of hydrogel digestion and antibacterial activity of an AMP. These results pave the way for the development of new macro‐bicyclic inhibitors of other chymotrypsin‐like family proteases, in particular, taking natural serine protease inhibitors as the templates.

## Conflict of interest

The authors declare no conflict of interest.

## Supporting information

As a service to our authors and readers, this journal provides supporting information supplied by the authors. Such materials are peer reviewed and may be re‐organized for online delivery, but are not copy‐edited or typeset. Technical support issues arising from supporting information (other than missing files) should be addressed to the authors.

Supporting Information
