# Supplementary material for: Diarylethene‐Based Photoswitchable Inhibitors of Serine Proteases
Source: Angew Chem Int Ed Engl. 2021 Aug 25;60(40):21789–94. doi: 10.1002/anie.202108847 (PMC8519022; doi:10.1002/anie.202108847)
Supplement: Supplementary file 1 — Supporting Information [file ANIE-60-21789-s001.pdf]

Angewandte Chemie
Eine Zeitschrift der Gesellschaft Deutscher Chemiker

Supporting Information

Diarylethene-Based Photoswitchable Inhibitors of Serine Proteases

Oleg Babii, Sergii Afonin, Christian Diel, Marcel Huhn, Jennifer Dommermuth, Tim Schober, Serhii Koniev, Andrii Hrebonkin, Alexander Nesterov-Mueller, Igor V. Komarov,* and Anne S. Ulrich**

anie_202108847_sm_miscellaneous_information.pdf

Author Contributions

O.B. Conceptualization: Equal; Formal analysis: Equal; Funding acquisition: Equal; Investigation: Equal; Methodology: Equal; Supervision: Equal; Validation: Equal; Visualization: Equal; Writing—original draft: Lead
S.A. Conceptualization: Equal; Formal analysis: Equal; Funding acquisition: Equal; Investigation: Equal; Methodology: Equal; Supervision: Equal; Validation: Equal; Visualization: Equal; Writing—review & editing: Equal
C.D. Formal analysis: Equal; Investigation: Equal
M.H. Formal analysis: Equal; Investigation: Equal
J.D. Formal analysis: Equal; Investigation: Equal
T.S. Formal analysis: Equal; Investigation: Equal; Visualization: Equal; Writing—review & editing: Equal
S.K. Formal analysis: Equal; Investigation: Equal; Visualization: Equal
A.H. Formal analysis: Equal; Investigation: Equal; Visualization: Equal
A.N. Formal analysis: Equal; Funding acquisition: Equal; Investigation: Equal; Methodology: Equal; Writing—review & editing: Equal
I.K. Conceptualization: Equal; Formal analysis: Equal; Methodology: Equal; Validation: Equal; Visualization: Equal; Writing—review & editing: Equal
A.U. Conceptualization: Equal; Formal analysis: Equal; Funding acquisition: Equal; Methodology: Equal; Project administration: Lead; Writing—review & editing: Equal.

Table of contents

1. Materials, reagents, and general procedures…… 2
2. Peptide synthesis…… 2
3. Preparation of peptides in the ring-closed photoforms…… 6
4. UV-Vis absorption spectra and photostationary state under UV irradiation…… 6
5. Photostability…… 10
6. Protease inhibition assays…… 11
7. Analysis of the proteolytic stability…… 19
8. Digestion of gelatin-based hydrogels…… 22
9. Regulation of the anti-bacterial action of peptide (BP100-RW)…… 22
10. Hydrogen/deuterium exchange rates…… 23
11. HPLC chromatograms…… 26
12. MALDI-TOF mass spectra…… 28
13. References…… 32

1. Materials, reagents, and general procedures

All fluorenylmethoxycarbonyl (Fmoc) N-protected amino acids, including the noncanonical Fmoc-L-propargylglycine (Fmoc-L-Pra-OH), Fmoc-L-γ-azidohomoalanine (Fmoc-L-Aha-OH), Fmoc-L-δ-azidoornithine (Fmoc-L-Orn(N_3)-OH), Fmoc-S-trityl-L-homocysteine (Fmoc-L-hCys(Trt)-OH), coupling reagents 2-(1H-benzotriazol-1-yl)-1,1,3,3-tetramethyluronium hexafluorophosphate (HBTU), 2-(1H-7-azabenzotriazol-1-yl)-1,1,3,3-tetramethyluronoium hexafluorphosphate (HATU), benzotriazole-1-yl-oxy-tris-pyrrolidino-phosphonium hexafluorophosphat (PyBOP), and the ligand tris(3-hydroxypropyltriazolylmethyl)amine (THPTA) for Cu-catalysed azide-alkyne cycloaddition reaction (CuAAC) were purchased from Iris Biotech. All other reagents and solvents were from Merck, ABCR, Fisher and used as received. The diarylethene-based building block in an N-Fmoc protected form (Fmoc-DAE-OH, 4-(2-(5-((((9H-fluoren-9-yl)methoxy)carbonyl)-L-prolyl)-2-methylthiophen-3-yl)cyclopent-1-en-1-yl)-5-methylthiophene-2-carboxylic acid) was synthesized according to published procedures.[S1]

Reversed-phase high-performance liquid chromatography (HPLC) was run on a Jasco LC-2000 series instrument with detection by UV absorbance using a diode array. For analytical studies, Vydac 218TP (C18) (4.6 mm × 250 mm, 10 μm) columns and, typically, a column temperature of 40°C, a flow rate of 1.5 mL/min, and a gradient elution with monotonous A:B gradients (slope: 1% B/min) have been employed. Eluent A: 90% H_2O, 10% acetonitrile (MeCN), 0.1 % trifluoroacetic acid (TFA), eluent B: 10% H_2O, 90% MeCN, 0.1 % TFA. For preparative HPLC, Vydac 218TP (C18) (22 mm × 250 mm, 10 μm) column, linear A:B gradients (slope: 2-4% B/min), a flow rate of 18 mL/min were used. The preparative process was thermostabilized to 40°C by preheating the eluent in a 10 mL injector loop submerged into a thermo-controlled water bath before entering the column. For some SFTI-1 analogues, a further elevated eluent temperature was necessary for high-quality purification.

Mass spectra for identification of peptides and hydrogen/deuterium exchange studies were recorded on a Bruker Autoflex III instrument using matrix-assisted laser-induced desorption/ionization (MALDI) coupled with a time-of-flight detection (MALDI-TOF-MS). MALDI depositions were prepared by co-crystallization with a matrix (α-cyano-4-hydroxycinnamic acid) from acidic (0.1% TFA) H_2O/MeCN solutions on a standard Bruker stainless-steel target.

2. Peptide synthesis

Peptides were synthesized by solid-phase peptide synthesis [S2], using Fmoc chemistry, and employing a Biotage Syro II automatic peptide synthesizer. Noncanonical amino acids and Fmoc-DAE-OH were coupled manually using 1.2 equiv of the Fmoc-protected compound, activated with 1.2 equiv of PyBOP, 1.2 equiv of HOBt, and 2.4 equiv of DIPEA (N,N-diisopropylethylamine) in DMF (dimethylformamide) (e.g., 0.5 mL DMF per 0.1 mmol of Fmoc-protected amino acid). Linear sequences were synthesized on a 2-chlorotrityl resin, preloaded with the first amino acid. Typical resin load was 0.5−0.8 mmol/g, and the reaction scale 0.1 mmol. For the standard amino acids, a double-coupling protocol with 4 equiv of Fmoc-protected amino acids, activated with HBTU/HOBt/DIPEA in DMF (20 min/coupling step), and Fmoc deprotection with 20% piperidine (20 min in DMF) were used. Fmoc-DAE-OH was coupled as the last building block in all linear sequences.

After completion of linear precursors, the resins were washed with DCM (dichloromethane) (3 times), hexane (2 times), and dried under vacuum. The precursors were cleaved from the resin without side-chain deprotection by a mixture of HFIP (1,1,1,3,3,3-hexafluoro-2-propanol) and DCM (1:3, v/v; 5 mL per 0.1 mmol load; 20 min). The solutions were filtered from the resin, and the solvent was removed on a rotary evaporator. The obtained crude oils were suspended in an H_2O/MeCN mixture (1:1, v/v) and lyophilized to remove HFIP traces. Then, linear precursors were cyclized without further purification in DCM (0.5 L per 0.1 mmol load) with an activator mixture of PyBOP (2 equiv) and HOBt (2 equiv) pre-dissolved in DMF (2 mL), followed by the addition of DIPEA (4 equiv). The reaction mixture was stirred for 24 h. The solvent was then concentrated on a rotary evaporator until approximately 50 mL of DCM was left, after which 200 mL water was added to the flask. Following the extraction, the water phase was discarded, and the DCM phase was evaporated to dryness.

The cyclized peptides' total deprotection was done with a cocktail containing TFA, H_2O, and TIS (triisopropylsilane) (93:5:2, v/v/v; 10 mL), incubation for 1 h at room temperature. During global deprotection, thiol-containing scavengers should be omitted for

peptides that bear azido groups to avoid unintentional reduction into amino functions.[S3] Afterward, the resin was filtered, the volatiles were removed on a rotary evaporator (30°C), and residual oils were lyophilized.

Crude peptides were dissolved in 10 mL of H_2O/MeCN mixture (2:1, v/v), filtered, and analyzed using analytical HPLC. Individual fractions were collected and analyzed by MALDI-TOF mass spectrometry. In all cases, the major component in crude materials was confirmed to be the target peptide. The peptides were then purified on a preparative HPLC, using typical gradients of 20-40% or 30−50% B.

After purification, the peptides with Cys and/or hCys residues were air-oxidized in 0.1 M ammonium bicarbonate solution (pH 8.0, for 24 h) to establish a disulfide bond and re-purified by HPLC. For the triazole bridge containing peptides, the formation of the azide-alkyne cross-link was done using the following protocol. 20-40 mg of pure peptide were dissolved in 20 mL of 50% *t*-butanol/H_2O mixture in a 50 mL flask. The peptide solution was then sealed with a septum and degassed by an argon purge for 10 min. In another 50 mL flask, a 10% t-butanol/H_2O mixture was prepared and degassed. In three separate Eppendorf® reaction tubes, samples of (i) 0.5 equiv (equivalents calculated in respect to the peptide) of $CuSO_4{\cdot}5H_2O$, (ii) 0.5 eqiuv. of THPTA (Tris((1-hydroxy-propyl-1H-1,2,3-triazol-4-yl)methyl)amine) ligand, and (iii) 1.5 equiv of sodium ascorbate were weighted out, each dissolved in 100 µL of degassed 10% t-butanol/H_2O mixture. The three components were then mixed and added to the peptide solution using 1 mL syringe with a long needle.[S4] The reaction was completed over 2-4 h. Analytical HPLC and MALDI-TOF-MS monitored its progress. After the reaction completion, an excess of ethylenediaminetetraacetic acid disodium salt (to complex the copper) was added, and the solution was lyophilized, followed by peptide purification by preparative HPLC.

Isolated yields of peptides obtained in the DAE ring-open form varied between 10 and 30 mg with purity ≥95% confirmed by analytical HPLC. These amounts were obtained by purification of some part of the synthesized crude materials, 40-100% depending on the purity. With the reaction scale 0.1 mmol used and molar mass of peptides in the range 1100 – 1850 g/mol, the theoretical yields are 110-185 mg, which is typically more than needed for the study. The purity of the obtained crude peptides after total deprotection roughly estimated by HPLC, are summarised in **Table S1**. The copper-catalysed click reaction and oxidative disulfide bond formation gave near quantitative yields.

Table S1. *Purity of the synthesized crude peptides after total TFA deprotection as estimated by HPLC analysis prior to purification.*

Peptide	PSS, %
**SFTI-1**	35
**SFTI-1i**	35
**S1n**	45
**S1i**	55
**S2**	25
**S3**	< 5
**S4**	35
**S5n**	65
**S5i**	65
**S6n**	60
**S6i**	45
**S7n**	25
**S7i**	25
**S8n**	25
**S9n**	35
**S10n**	40
**S10i**	45
**S11i**	40
**S12i**	20
**S5F**	40

Synthesized peptides and their properties are listed in **Table S2**, and chemical structures are shown in **Figure S1**.

Table S2. *The nomenclature, sequences, types of cross-linker, analytical data for **SFTI-1** and its analogues.*

Peptide	Sequence[a]	Bridge (residues, length, [atoms])	Mass calculated, [m/z]	Mass measured, [m/z]
**SFTI-1**	$_{cyclo}$[-GRCTKSIPPICFPD-]	Cys-Cys, 4	1513.8	1513.7
**SFTI-1i**	$_{cyclo}$[-GFCTKSIPPICRPD-]	Cys-Cys, 4	1513.8	1513.7
**S1n**	$_{cyclo}$[-**1**-RCTKSIPPICF-]	Cys-Cys, 4	1628.1	1627.7
**S1i**	$_{cyclo}$[-**1**-FCTKSIPPICR-]	Cys-Cys, 4	1628.1	1627.6
**S2**	$_{cyclo}$[-**1**-RATKSIPPIAF-]	no	1565.9	1565.7
**S3**	$_{cyclo}$[-**1**-ATKSIPPIA-]	no	1262.5	1262.3
**S4**	$_{cyclo}$[-**1**-TKSIPPI-]	no	1120.4	1120.4
**S5n**	$_{cyclo}$[-**1**-RCTKSIPPI-hCys-F-]	hCys-Cys, 5	1642.0	1641.7
**S5i**	$_{cyclo}$[-**1**-FCTKSIPPI-hCys-R-]	hCys-Cys, 5	1642.0	1641.7
**S6n**	$_{cyclo}$[-**1**-R-*Pra-TKSIPPI-*Aha-F-]	Pra-Aha*, 6	1645.0	1644.7
**S6i**	$_{cyclo}$[-**1**-F-*Pra-TKSIPPI-*Aha-R-]	Pra-Aha*, 6	1645.0	1644.7
**S7n**	$_{cyclo}$[-**1**-R-*Pra-TKSIPPI-*Orn(N_3)-F-]	Pra-Orn(N_3)*, 7	1659.0	1658.8
**S7i**	$_{cyclo}$[-**1**-F-*Pra-TKSIPPI-*Orn(N_3)-R-]	Pra-Orn(N_3)*, 7	1659.0	1658.8
**S8n**	$_{cyclo}$[-**1**-VRCTKSIPPICF-]	Cys-Cys, 4	1727.2	1727.7
**S9n**	$_{cyclo}$[-**1**-RCTKSIPPICFL-]	Cys-Cys, 4	1741.2	1740.7
**S10n**	$_{cyclo}$[-**1**-VRCTKSIPPICFL-]	Cys-Cys, 4	1840.3	1839.7
**S10i**	$_{cyclo}$[-**1**-VFCTKSIPPICRL-]	Cys-Cys, 4	1840.3	1839.8
**S11i**	$_{cyclo}$[-**1**-VF-*Pra-TKSIPPI-*Aha-RL-]	Pra-Aha*, 6	1857.2	1856.8
**S12i**	$_{cyclo}$[-**1**-VF-*Pra-TKSIPPI-*Orn(N_3)-RL-]	Pra-Orn(N_3)*, 7	1871.2	1870.8
**SFTI-5F**	$_{cyclo}$[-GRCTFSIPPICFPD-]	Cys-Cys, 4	1532.0	1532.4
**S5F**	$_{cyclo}$[-**1**-VRCTFSIPPICFL-]	Cys-Cys, 4	1858.3	1857.8

[a] Canonical L amino acids are shown in one-letter code. The stabilizing bridge-forming amino acids are indicated with three- and four-letter abbreviations: hCys – L-homocysteine; Aha – L-δ-azidohomoalanine; Orn(N_3) – L-δ-azidoornithine; **1** – diarylethene-based photoswitching building block; disulfide cross-linked residues are underlined; the 1,4-disubstituted 1,2,3-triazole bridges between the azido and alkyne bearing side-chains are indicated with an asterisk sign (*).

Figure S1. *The structures of SFTI-1 and its analogues that were synthesized in this study.*

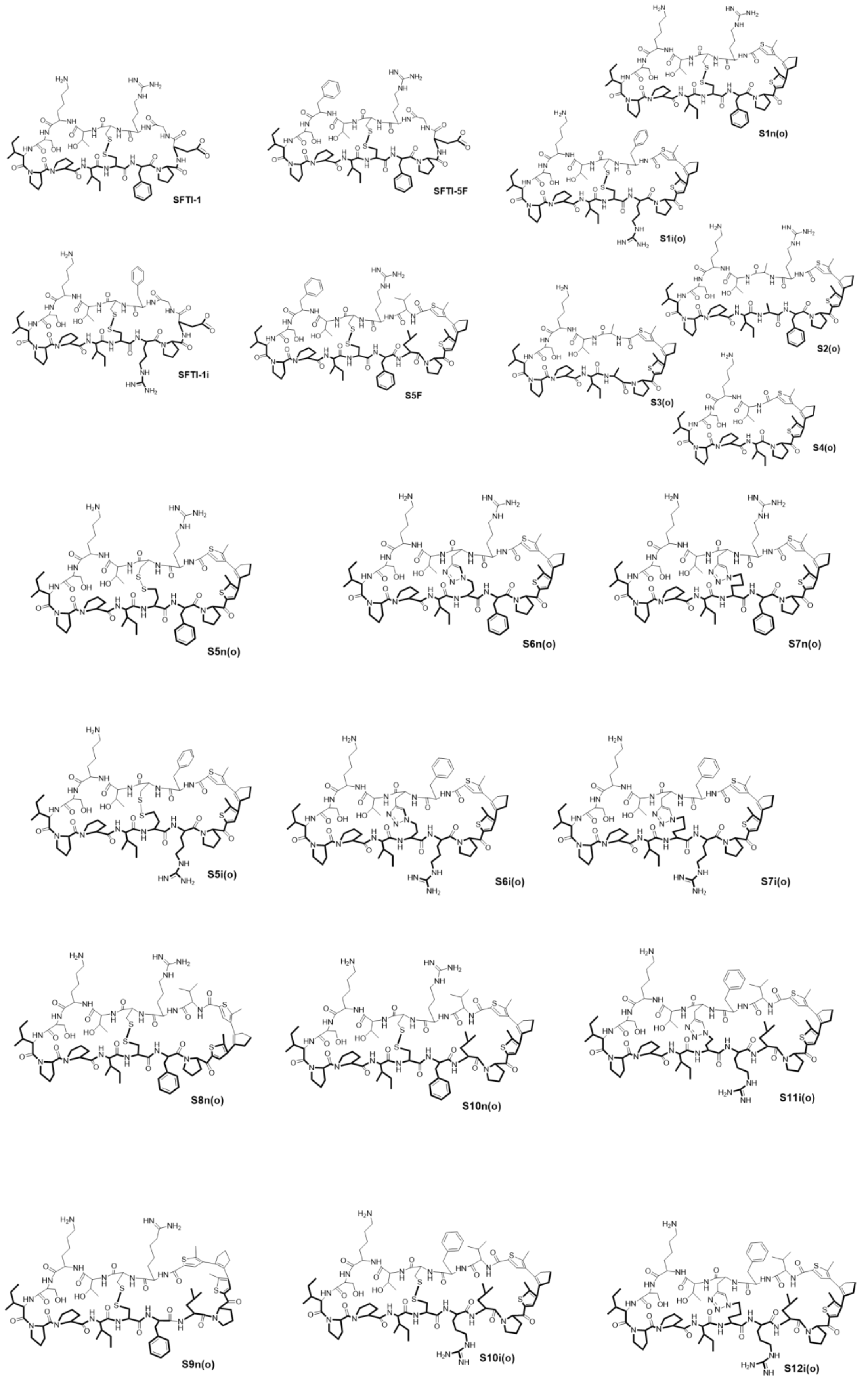

3. Preparation of peptides in the ring-closed photoforms

All photoswitchable peptides were prepared in 100% pure ring-closed photoform by HPLC separation of an equilibrium mixture of the two photoforms, ring-open and ring-closed, obtained after UV irradiation. It has been reported that the diarylethene-based photoswitchable peptides with a cyclic structure in chaotropic solutions, such as concentrated urea, convert to the ring-closed photoform with higher efficiency compared to pure water or organic solvents. This effect was explained by the loosening of intramolecular hydrogen bonds in chaotropic solutions.[S1] Typically, 10 mg of a peptide in the ring-open form (with established disulfide bond or triazole linkage, if present) were dissolved in 10 mL of a freshly prepared saturated urea solution acidified by addition of 100 µL of 1N HCl. The solution was placed in a 500 mL conical flask and degassed an argon purge for 5 min. The degassed solution was then irradiated in the conical flask under constant stirring and argon atmosphere with UV light using a LUMATEC® light source (Superlite® S04 model) equipped with an optic cable (THORLABS® LLG0538-8 liquid light guide, 5 mm core diameter). The irradiation conditions were: spectral range - 320−400 nm, light power - 2.1 W, power density - 10.5 mW/cm^2, 15 cm distance from the tip of the light guide to the solution surface, 10 min exposure time. This procedure yielded 60-80% conversion to the ring-closed form, except for the peptides **S5n**, **S5i**, **S8n**, **S9n,** which had ~20% of the ring-closed form in the photostationary state, and the peptides **S1n**, **S1i**, **S4**, which were not photoswitchable (0% conversion to the ring-closed form). After the UV irradiation, the urea solutions were diluted with the HPLC eluent A (20 mL). The pure ring-closed forms were separated by preparative HPLC (non-converted ring-open forms were recovered). Chromatographic fractions were lyophilized in the light-protected flasks (wrapped with aluminum foil) and stored in the dark as dry powders at −80°C.

4. UV-Vis absorption spectra and photostationary state under UV irradiation

The UV-Vis absorption spectra were measured in water at a peptide concentration of 20 mM at 22 °C. The solution was purged with argon to remove dissolved oxygen. Measurements were made in a quartz cuvette with a pathlength 10 mm using Ultrospec™ 8000 spectrophotometer. For each peptide, the ring-closed form spectrum was first recorded. Then, the sample was irradiated with visible light for 1 min (LUMATEC® light source, 570 nm wavelength filter, power density 143 mW/cm^2) and a spectrum of the ring-open form was obtained. To characterize the photostationary state and the kinetics of conversion from the ring-open to ring-closed forms, the sample was irradiated with UV light (LUMATEC® light source, spectral range of 320-400 nm, power density 167 mW/cm^2) and absorption spectra were recorded at different time intervals. The spectra and conversion kinetics are shown in **Figure S2**; the corresponding photoconversions in the photostationary state are summarized in **Table S3**. It is important to note that the PSSs measured in these experiments differ slightly from the conversions obtained during the preparation of the pure ring-closed forms (see Section 3.), because these experiments were performed in a saturated urea solution to break hydrogen bonds and at higher peptide concentrations.

Figure S2. *UV-Vis absorption spectra of studied peptides measured in water at 20 mM concentration in quartz cuvette with 10 mm pathlength at 22°C. A tripeptide containing the photoswitch (Ac-Ala-**1**-Ala-NH$_2$) was included for comparison. Photostationary states (PSS) were identified as the maximum conversions reached. The left plots show absorption spectra normalized at 575 nm for the ring-closed forms. The right plots show the dependence of conversion on time of UV irradiation.*

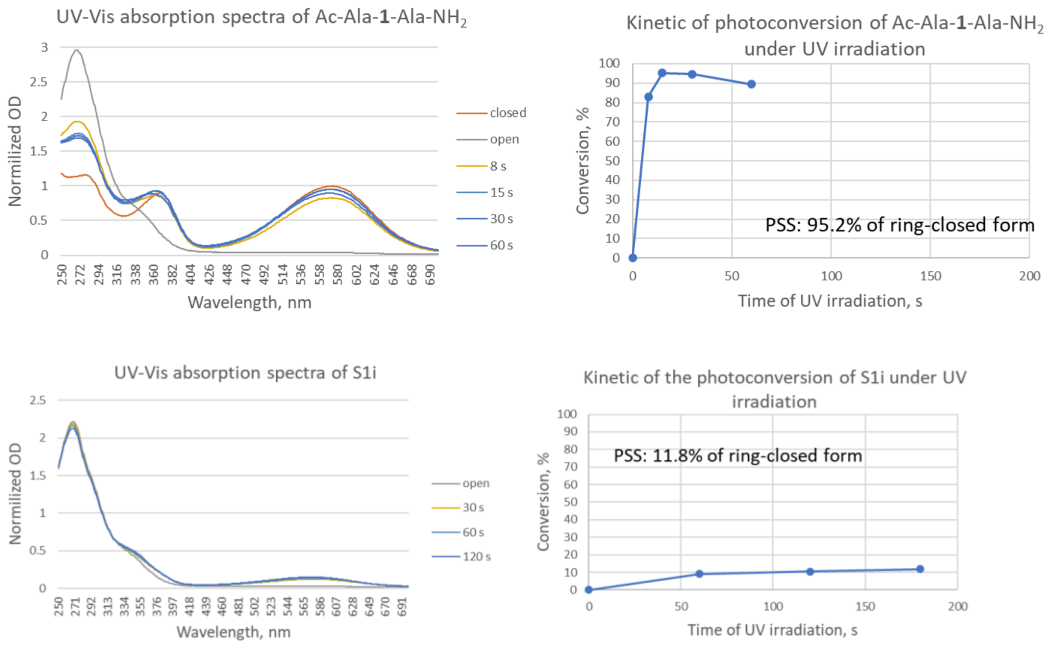

Figure S2 *(continuation)*

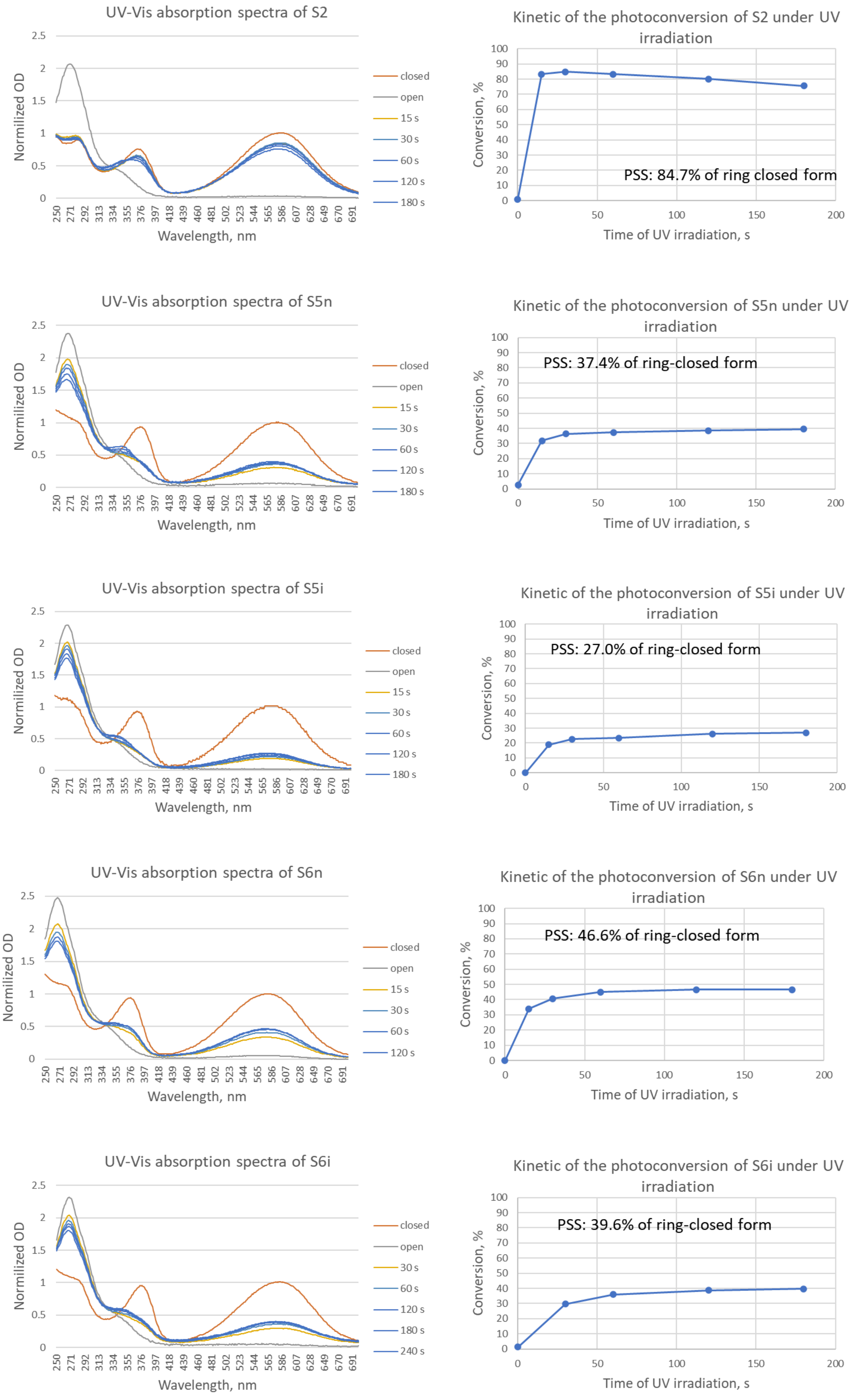

Figure S2 *(continuation)*

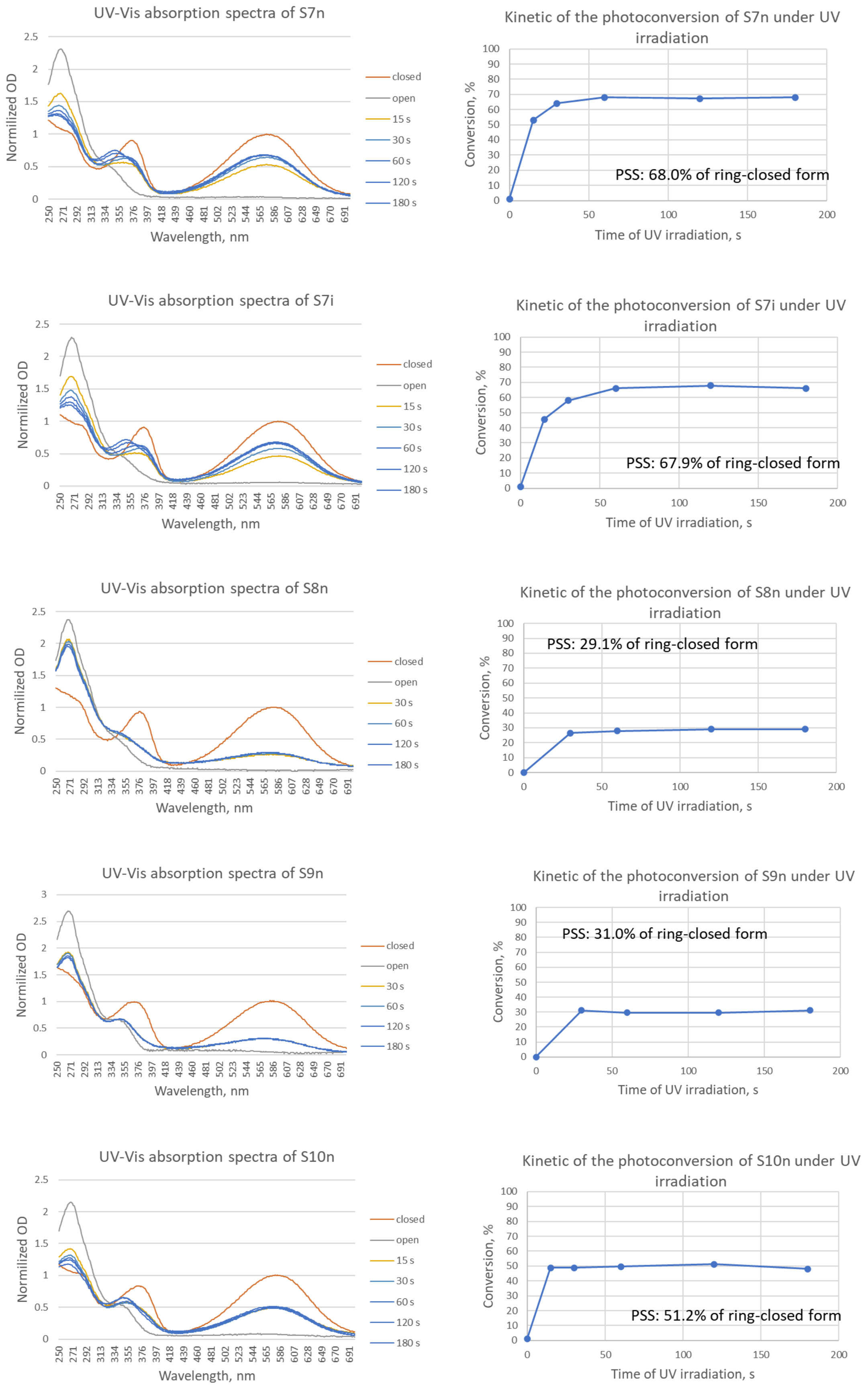

Figure S2 *(continuation)*

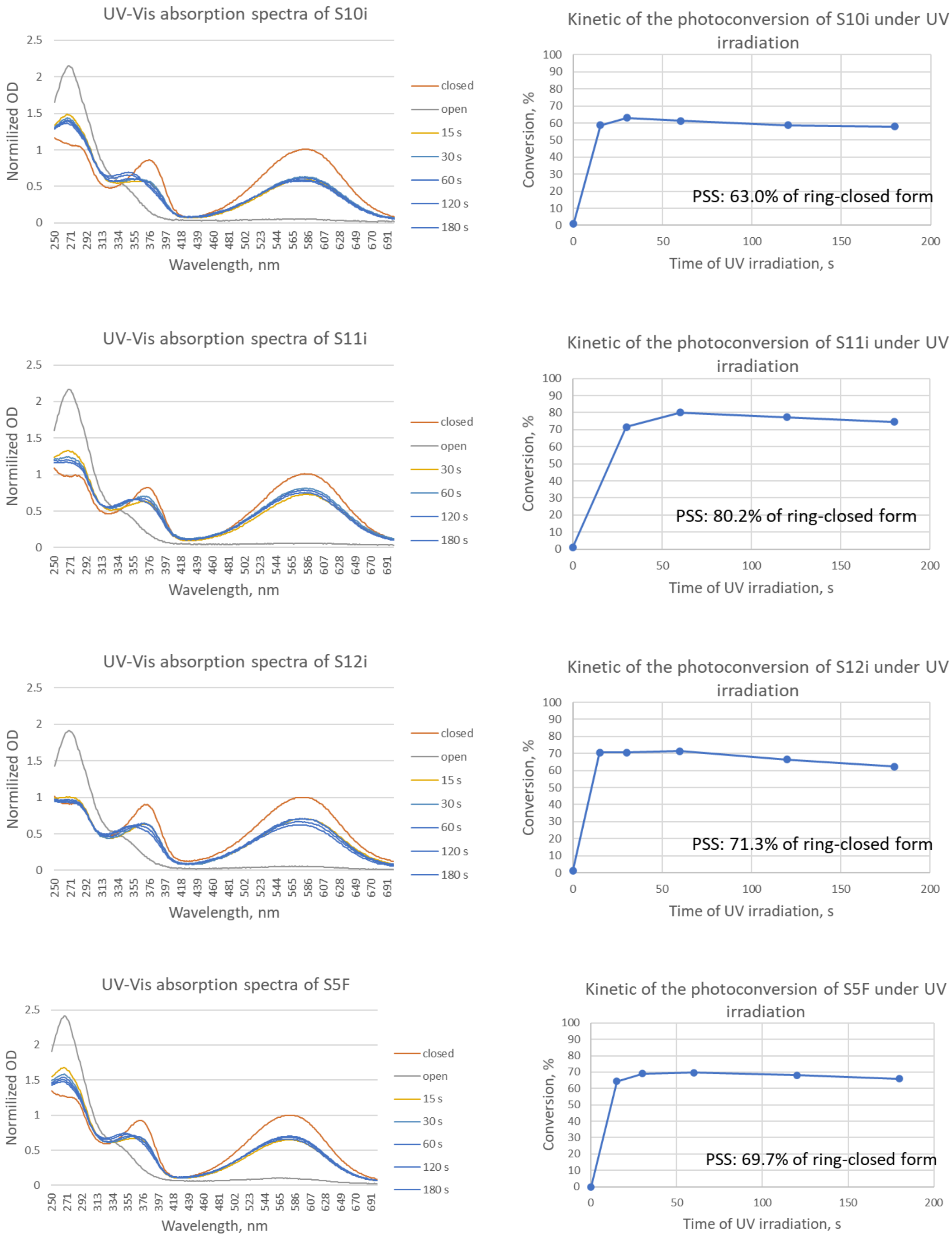

Table S3 *Photostationary states (PSS) for the studied peptides in deoxygenated water at 22 °C determined as the maximal conversion of the ring-open form to ring-closed form under continuous UV irradiation (spectral range of 320-400 nm).*

**Peptide**	**PSS, %**
**Ala-1-Ala**	95.2
**S1n**	< 10*
**S1i**	11.8*
**S2**	84.7
**S3**	< 10*
**S4**	< 10*
**S5n**	37.4
**S5i**	27.0
**S6n**	46.6
**S6i**	39.6
**S7n**	68.0
**S7i**	67.9
**S8n**	29.1
**S9n**	31.0
**S10n**	63.0
**S10i**	51.2
**S11i**	80.2
**S12i**	71.3
**S5F**	69.7

* As was determined by RP-HPLC analysis. Data not shown.

5. Photostability

The photostability in repeated cycles of reversible photoisomerization was studied on three selected compounds (linear three-peptide Ac-Ala-**1**-Ala-NH_2, lacking the bridge peptide **S2**, and the one with the biggest change in activity upon photoisomerization **S10i**). The samples were prepared in the same manner as for UV-Vis absorption characterization, namely at 20 mM in deoxygenated water. The UV irradiation was done at spectral range of 320-400 nm, 167 mW/cm^2 for 15 s; the visible light irradiation at 570 nm, 143 mW/cm^2 for 60 s (both using LUMATEC® light source). The results on photostability are shown in **Figure S3**. All three compounds showed a great fatigue resistance with little degradation after 15 cycles of conversion.

Figure S3. *Photostability study of the three selected peptides at 20 mM in deoxygenated water at 22°C in 15 cycles of reversible photoisomerization with visible (570 nm, 143 mW/cm^2 for 60 s) and UV light (320-400 nm, 167 mW/cm^2 for 15 s). The left plots show absorption spectra after each 5 cycles (normalized at 575 nm for ring-closed forms). The right plots show the % of ring-closed form measured as absorbance at 575 nm.*

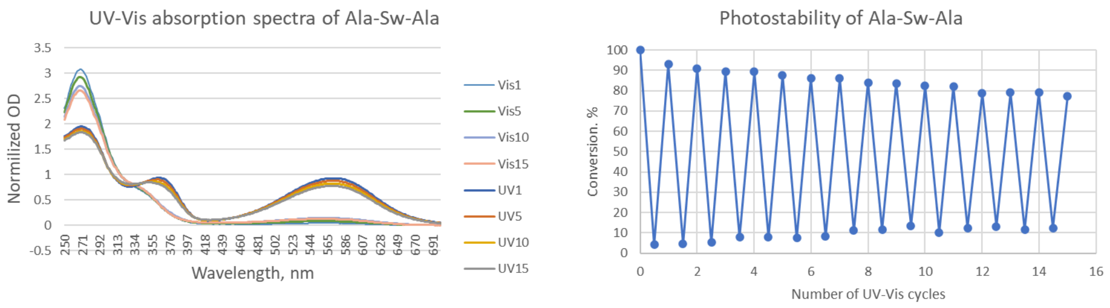

Figure S3 *(continuation)*

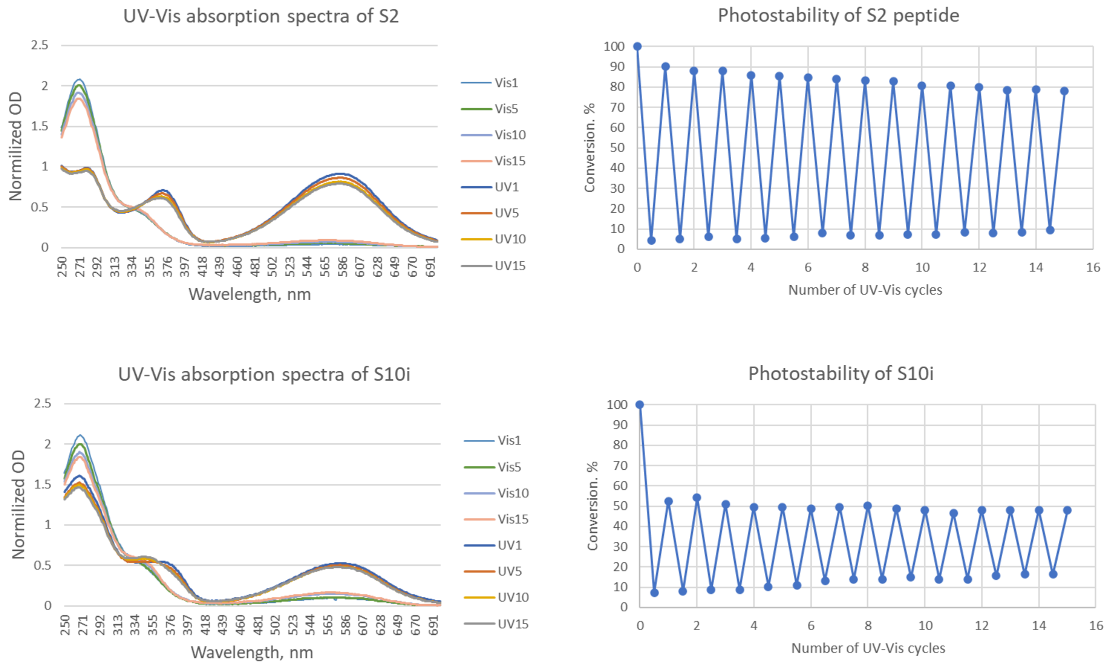

6. Protease inhibition assays

The inhibition of trypsin and α-chymotrypsin was investigated by two methods. First, the inhibition was determined at a fixed enzyme and substrate concentration, whereas the inhibitor concentration varied. By plotting the protease activity against an inhibitor's concentration, IC_{50} (the half-maximal inhibitory concentration) was determined. This method allows an estimation of inhibitory activity, but it does not allow an accurate determination of the inhibitory constant (K_i) for the potent inhibitors. With the second method, using Lineweaver–Burk analysis (see below), the K_i values for all peptide inhibitors were determined with high accuracy.

All measurements were performed with *p*-nitroaniline-based chromogenic substrates (λ_{abs} = 405 nm) in 96-well microtiter plates using Epoch^{TM} 2 microplate spectrophotometer. For trypsin measurements the Tris buffer (50 mM Tris-HCl, 25 mM $CaCl_2$, 0.005% Tween® 20, pH 8,2) was used. Enzyme aliquots with a concentration of 4.3 µM (0.1 mg/mL, MW = 23.3 kDa) in 1 mM HCl were prepared and stored at -80°C. For the measurements, a fresh 0.92 µM trypsin solution was prepared from the storage aliquot and kept on ice. A stock solution of the substrate BAPNA (Nα-Benzoyl-DL-arginine 4-nitroanilide hydrochloride) at the concentration of 50 mM in aqueous dimethyl sulfoxide (DMSO) (100 mg of BAPNA in 4.6 mL of DMSO:water mixture 1:1) was prepared and stored at RT. The peptide solutions at the concentration of 100 µM, which was further diluted to 10, 1, or 0.1 µM if required, were prepared in Milli-Q® deionized ultrapure water.

For the determination of IC_{50}, a double dilution series of inhibitors were prepared in a 96-well plate (12 concentrations, two repeats for each concentration, 200 µL of Tris buffer in each well). Subsequently, a trypsin solution (20 µL, 150 nM) was added to each well with the tested peptide and to the control row. Solutions in each well were intensely mixed using a multichannel pipette (from lower concentration to higher). After a pre-incubation period (5 min), the substrate BAPNA was added to each well (20 µL, 25 mM). The solutions were thoroughly mixed with the multichannel pipette, and the kinetics of substrate hydrolysis was recorded with an interval of 1 min between measurements over 20 min. The total volume in each well was 240 µL, final trypsin and BAPNA concentrations were 12.5 nM and 2.08 mM, respectively. The mean values of the two repeats of the slope of absorption vs. time curves were calculated. The activities at different inhibitor concentrations were then calculated by taking the control curves' slope values (no inhibitor) as the 100% enzyme activity. The inhibition activity IC_{50} against α-chymotrypsin (α-chymotrypsin from bovine pancreas type II) was measured in the same way except that a different substrate, Ac-EAPF-pNA (Ac-Glu-Ala-Pro-Phe-pNA), was used (20 µL, 16 mM). The obtained IC_{50} curves are summarized in **Figure S4**.

SUPPORTING INFORMATION

***Figure S4** Trypsin and α-chymotrypsin inhibitory activities of studied peptides as a function of inhibitor concentration and the respective IC_{50} values measured at a fixed enzyme (12.5 nM) and substrate (2.08 mM for BAPNA and 1.33 mM for Ac-EAPF-pNA) concentrations. The measurement for **SFTI-1** is repeated four times.*

The anti-trypsin activity SFTI-1

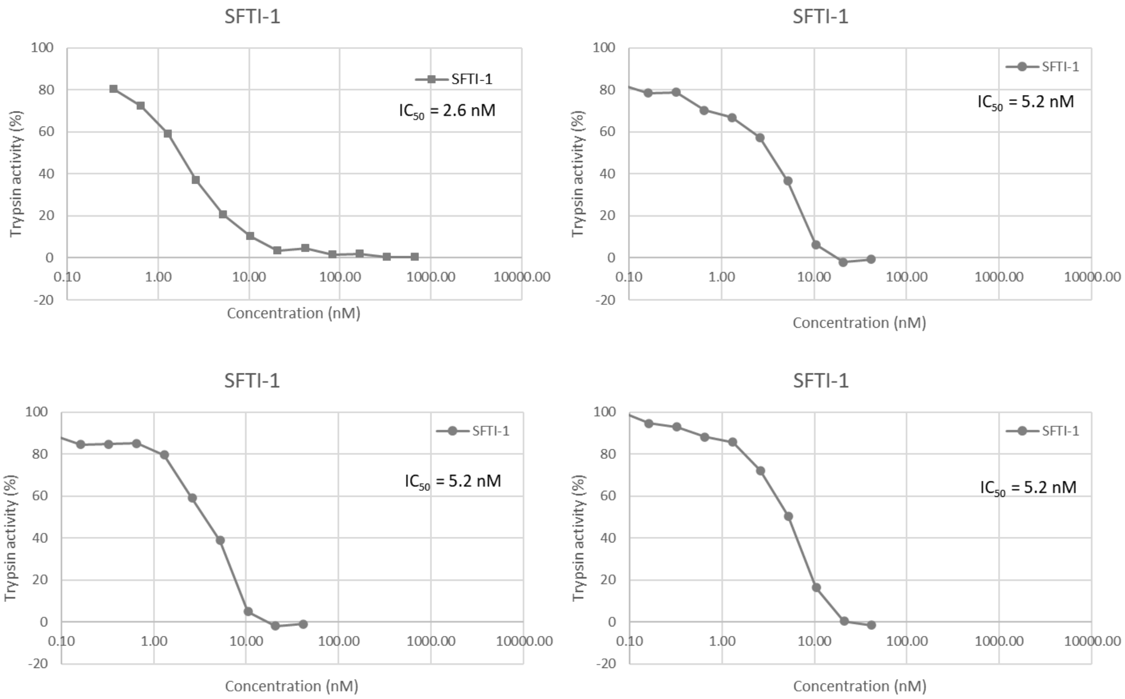

The anti-trypsin activity analogues

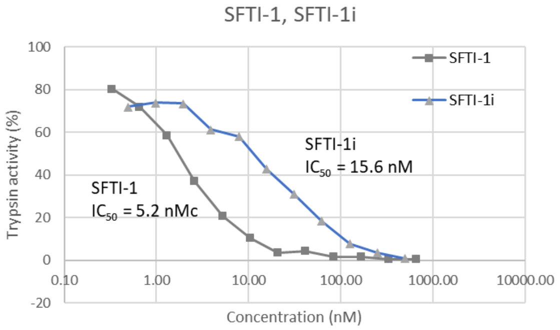

Figure S4 *(continuation)*

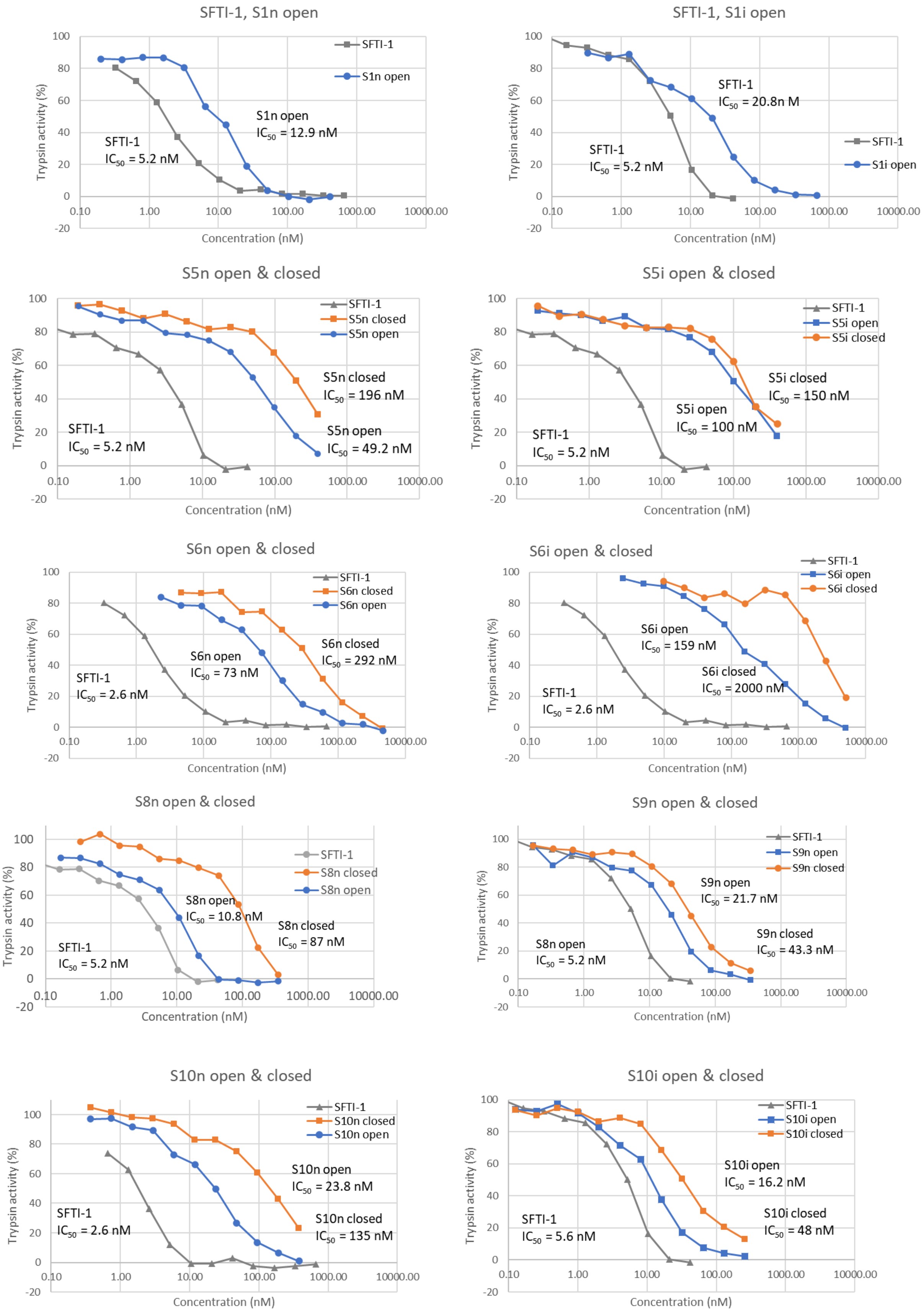

Figure S4 *(continuation)*

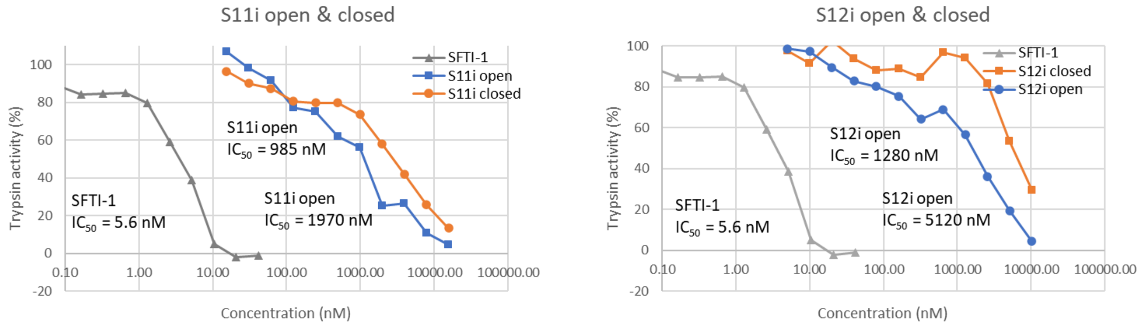

The anti- α-chymotrypsin activity

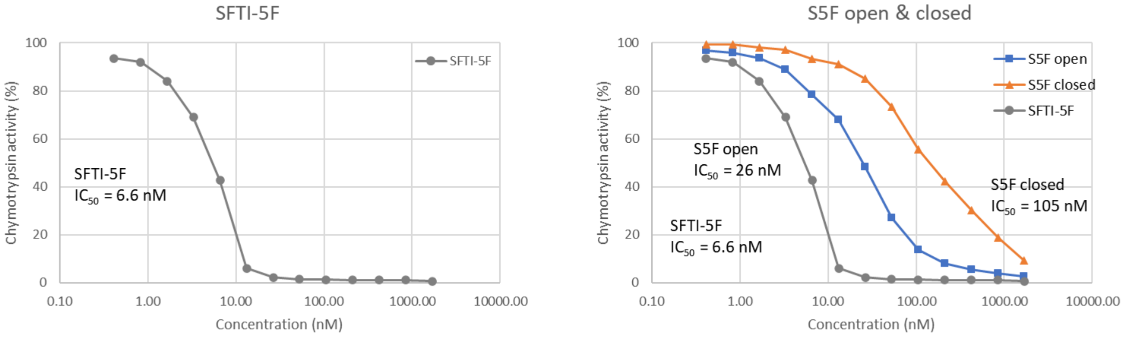

The selectivity of S5F and S10n

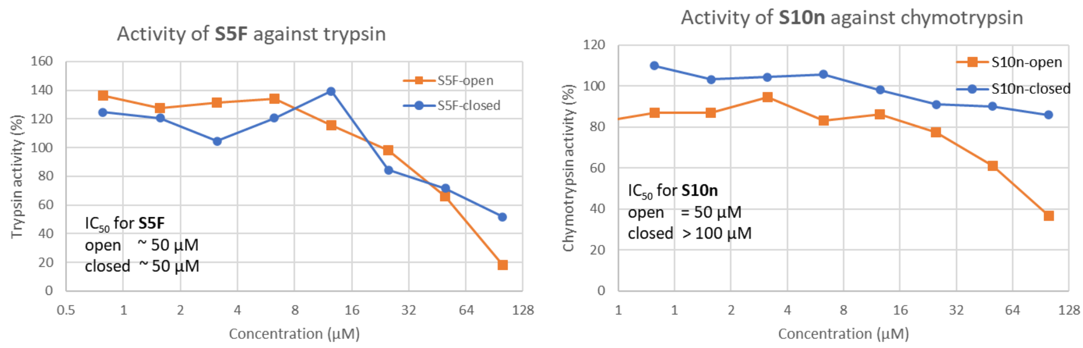

Inhibitory constants K_i were determined by Lineweaver-Burk (LWB) double-reciprocal analysis according to a published protocol.[S5] Briefly, a fresh inhibitor/enzyme mixture was prepared in a glass vial and distributed to 2x6 wells (200 µL in each well, enzyme concentration after dilution 12 nM). An enzyme without an inhibitor was added to the control wells. Aliquots of varying substrate stock solutions were added with a multichannel pipette to each well. For trypsin inhibition experiments, BAPNA (20 µL per well of 20, 22.7, 26, 29.8, 34, 40 mM) and for α-chymotrypsin Ac-EAPF-pNA (20 µL per well of 16, 12.48, 10, 8, 6.4, 5.44 mM) were used, respectively.

Well contents were thoroughly mixed using a multichannel pipette, and a plate reader was used to monitor the hydrolysis kinetics (sampling interval of 1 min over 20 min). For each peptide inhibitor, an appropriate LWB analysis concentration (a concentration inhibiting the enzyme in a range between 30-70%) was taken based on the prior determined IC_{50} values. Optimal parameters for LWB analysis (concentration) and obtained K_i values are given in **Table S4**. The respective LWB plots are shown in **Figure S5**.

Table S4 *Trypsin and α-chymotrypsin inhibition IC_{50} values, concentrations selected for LWB analysis, and K_i values for all studied peptide inhibitors as determined by LWB analysis.*

Peptide	IC_{50}, nM	Concentrations used for LWB analysis, nM	K_i, nM
The trypsin inhibition			
**SFTI-1**	4.5	4	3.4±0.2
**SFTI-1i**	15.6	20	12.5±0.8
**S1n** open	12.9	20	4.9±0.3
**S1i** open	20.8	20	13.4±1.4
**S5n** open	49.2	85	95±6
**S5n** closed	196	350	231±14
**S5i** open	100	175	102±14
**S5i** closed	150	175	182±16
**S6n** open	73	130	135±8
**S6n** closed	292	520	1120±109
**S6i** open	159	260	441±43
**S6i** closed	2000	3000	7700±600
**S7n** open	38	65	63.4±4.4
**S7n** closed	900	1500	2200±214
**S7i** open	105	200	263±30
**S7i** closed	3375	6250	7220±360
**S8n** open	10.8	27	5.2±0.4
**S8n** closed	87	176	125±8
**S9n** open	21.7	40	8.3±0.6
**S9n** closed	43.3	80	55±3.8
**S10n** open	23.8	20	17.5±0.7
**S10n** closed	135	350	181±7
**S10i** open	16.2	20	5.1±0.4
**S10i** closed	48	85	44.8±2.2
**S11i** open	985	800	1720±130
**S11i** closed	1970	3100	5370±420
**S12i** open	1280	1050	2440±215
**S12i** closed	5110	4200	12400±900
**S5F** open	~ 50 000	-	-
**S5F** closed	~ 50 000	-	-
The α-chymotrypsin inhibition			
**SFTI-5F**	6.6	7	2.8±0.1
**S5F** open	26	26	5.8±0.1
**S5F** closed	105	105	122±1
**S10n** open	50 000	-	-
**S10n** closed	> 100000	-	-

Figure S5 *Experimental LWB plots for the studied peptides.*

The trypsin inhibition

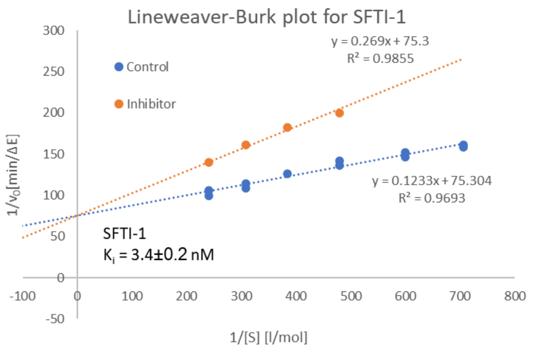

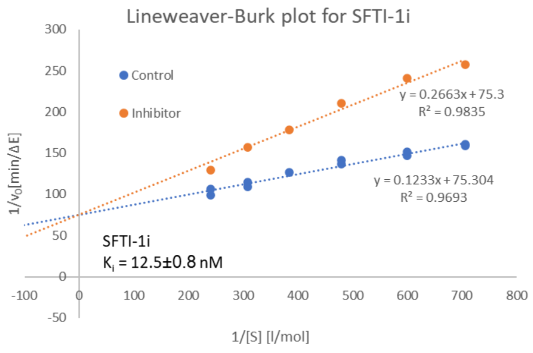

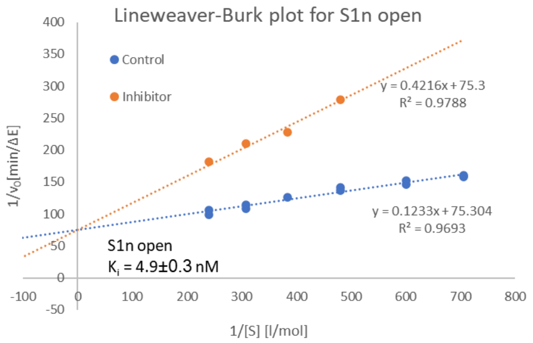

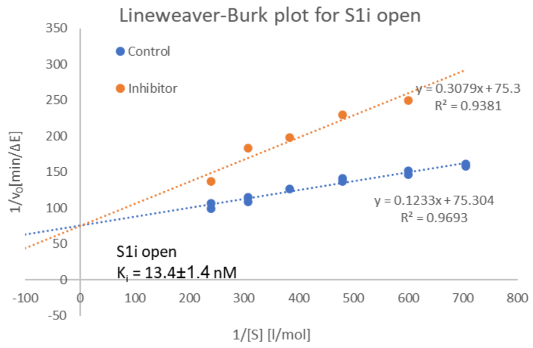

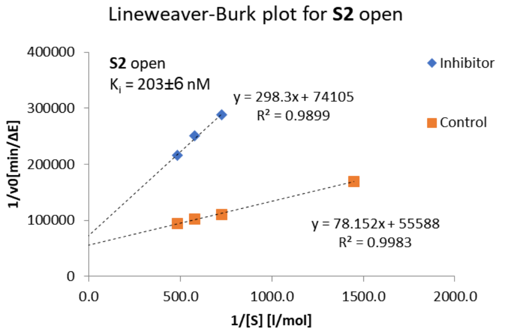

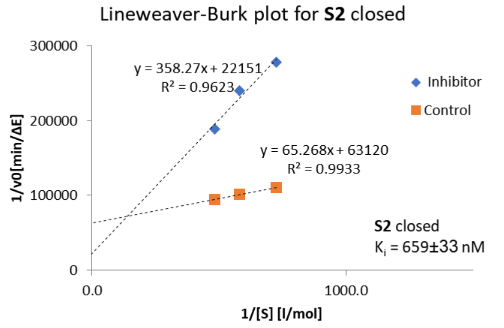

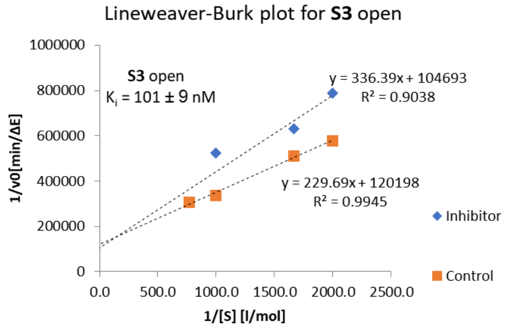

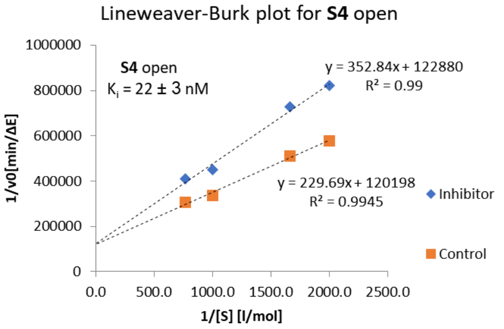

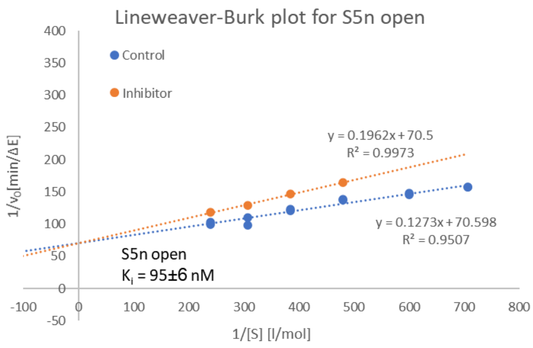

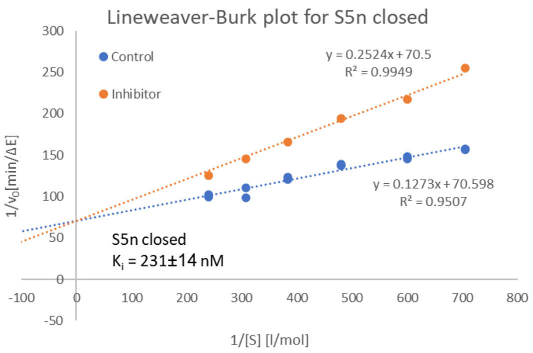

Figure S5 *(continuation)*

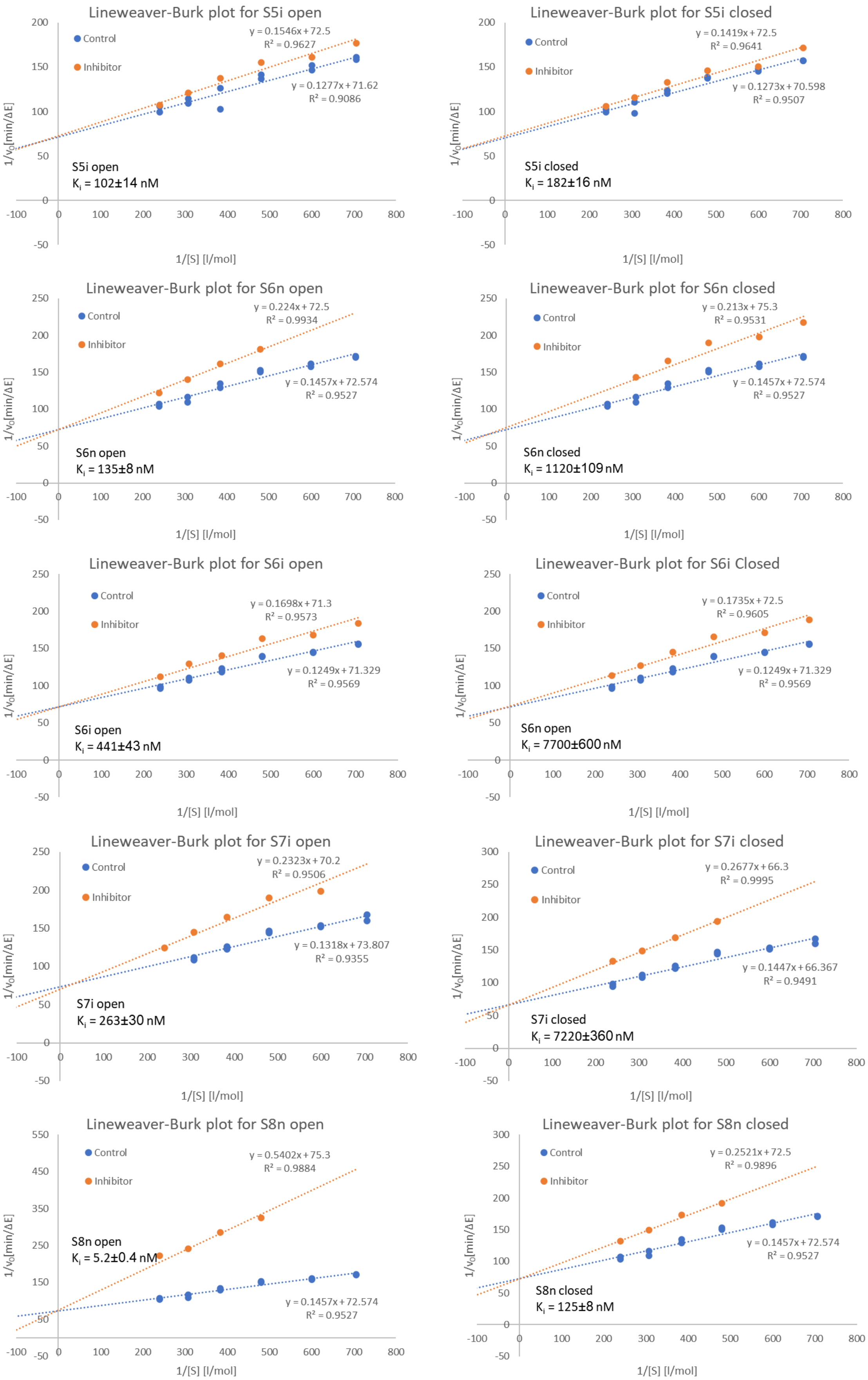

Figure S5 *(continuation)*

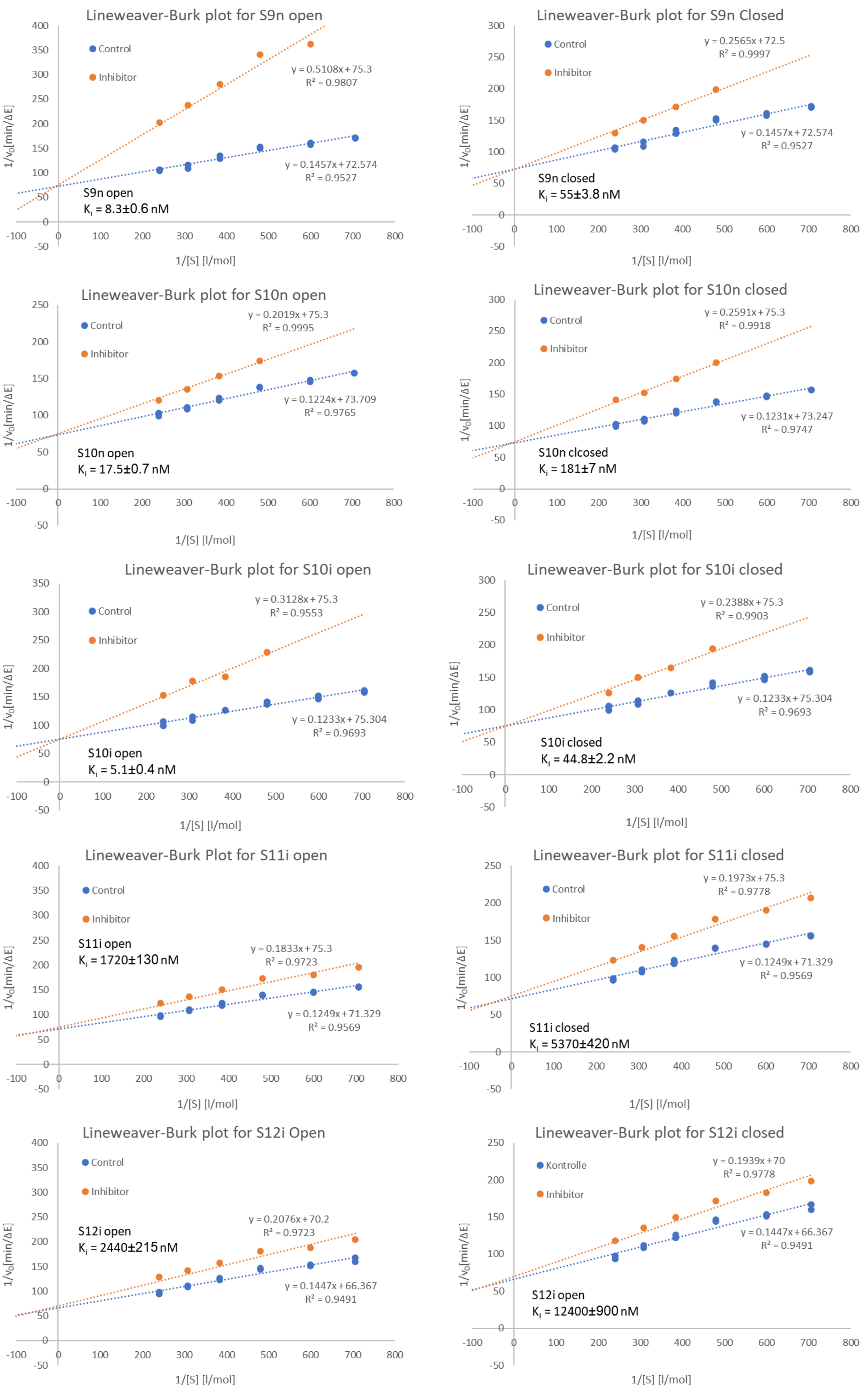

Figure S5 *(continuation)*

The α-chymotrypsin inhibition

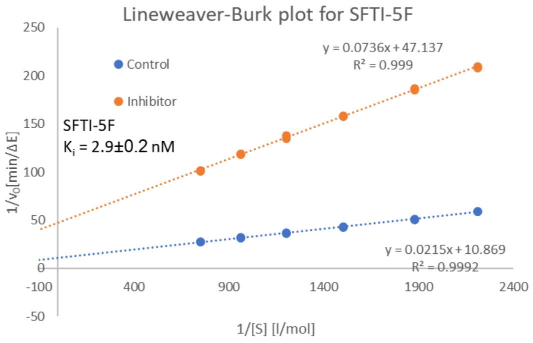

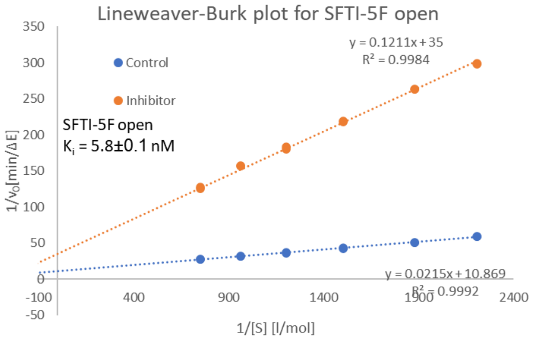

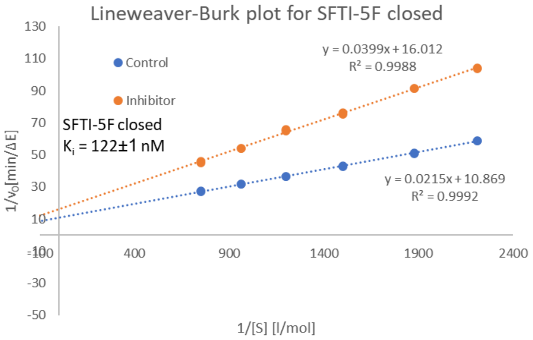

7. Analysis of the proteolytic stability

Proteolytic stability of the peptides was characterized by determining hydrolysis rates (k_H) by the action of trypsin in a 50 mM Tris–HCl buffer, pH 8.2, supplemented with 25 mM $CaCl_2$ and 0.005% Tween®20 at 22°C. The peptides at a concentration of 50 µM were mixed with trypsin (0.5 µM) in a 2 mL total volume. At selected time intervals, 70 µL samples of the mixture were taken, quenched by addition 130 µL 2% aqueous formic acid, and immediately analyzed by analytical HPLC. The k_H values were calculated from the time-dependent data assuming zero-order kinetics (zero-order kinetics applies due to the high substrate/enzyme ratio of 100 to 1). For the fitting the linear part of the hydrolysis curve was taken excluding the last time point in which the entire peptide inhibitor was hydrolyzed.[S6] **Table S5** summarizes the obtained k_H values.

A general tendency was noted when comparing the hydrolysis profiles of the original and "inverted" analogues. Original sequence analogues could hydrolyze at two positions: at the Lys (P1 position) of the inhibitory loop and after the stabilizing loop's arginine residue (P4 position).[S6] The ultimate product (i.e., cleaved at both positions) had, therefore, a MALDI-TOF-MS detectable mass increase of +36 Da. However, during hydrolysis of "inverted" analogues, only a single product, +18 Da, resulting from the single cleavage at the P1 position was observed. The arginine residue's amide bond's stability in the "inverted" position presumably increased due to a steric hindrance in the bridge's proximity. This behavior is exemplified by chromatograms of **S5n** ring-open form and **S5i** ring-open form in **Figure S6**; chromatography data are collected in **Figure S7**.

Figure S6 *Selected HPLC chromatograms from the* ***S5n*** *open (left panel) and* ***S5i*** *open (right panel) undergoing trypsinolysis, obtained when determining K_H. The identity of each peak was confirmed by MALDI-TOF-MS. Detection – 220 nm, hydrolysis time is indicated at each chromatogram.*

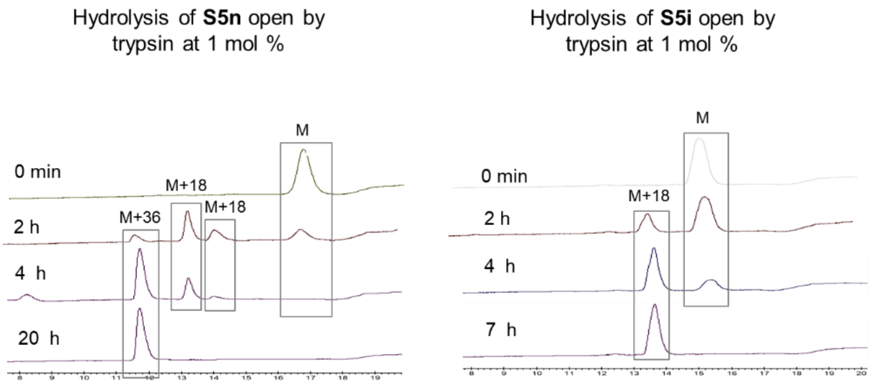

Table S5 *Hydrolysis rates of all studied peptides in both photoforms.*

Peptide	k_H, $M^{-1}\cdot s^{-1}$	
	ring-open	*ring-closed*
**SFTI-1**	$2.0\pm0.1\cdot10^{-5}$	-
**SFTI-1i**	$2.0\pm0.2\cdot10^{-5}$	-
**S1n**	$7.8\pm0.2\cdot10^{-5}$	-
**S1i**	$8.3\pm0.3\cdot10^{-5}$	-
**S5n**	$2.5\pm0.1\cdot10^{-3}$	$3.4\pm0.5\cdot10^{-2}$
**S5i**	$2.9\pm0.1\cdot10^{-3}$	$5.0\pm0.8\cdot10^{-3}$
**S6n**	$6.7\pm0.9\cdot10^{-3}$	$2.5\pm0.8\cdot10^{-2}$
**S6i**	$1.9\pm0.1\cdot10^{-2}$	$1.1\pm0.2\cdot10^{-1}$
**S7n**	$2\pm0.2\cdot10^{-3}$	$6.1\pm1.2\cdot10^{-2}$
**S7i**	$7.5\pm0.2\cdot10^{-3}$	$5.5\pm1\cdot10^{-1}$
**S8n**	$1.3\pm0.7\cdot10^{-4}$	$5.3\pm1.4\cdot10^{-4}$
**S9n**	$1.5\pm0.3\cdot10^{-4}$	$1.3\pm0.1\cdot10^{-3}$
**S10n**	$2.3\pm0.2\cdot10^{-4}$	$3.6\pm0.3\cdot10^{-3}$
**S10i**	$5.3\pm0.4\cdot10^{-5}$	$2.2\pm0.3\cdot10^{-4}$
**S11i**	$2.8\pm0.3\cdot10^{-2}$	$9.1\pm1\cdot10^{-2}$
**S12i**	$1.4\pm0.3\cdot10^{-2}$	$4.2\pm0.4\cdot10^{-2}$

Figure S7 *Kinetics of trypsinolysis of the studied peptides in both photoforms.*

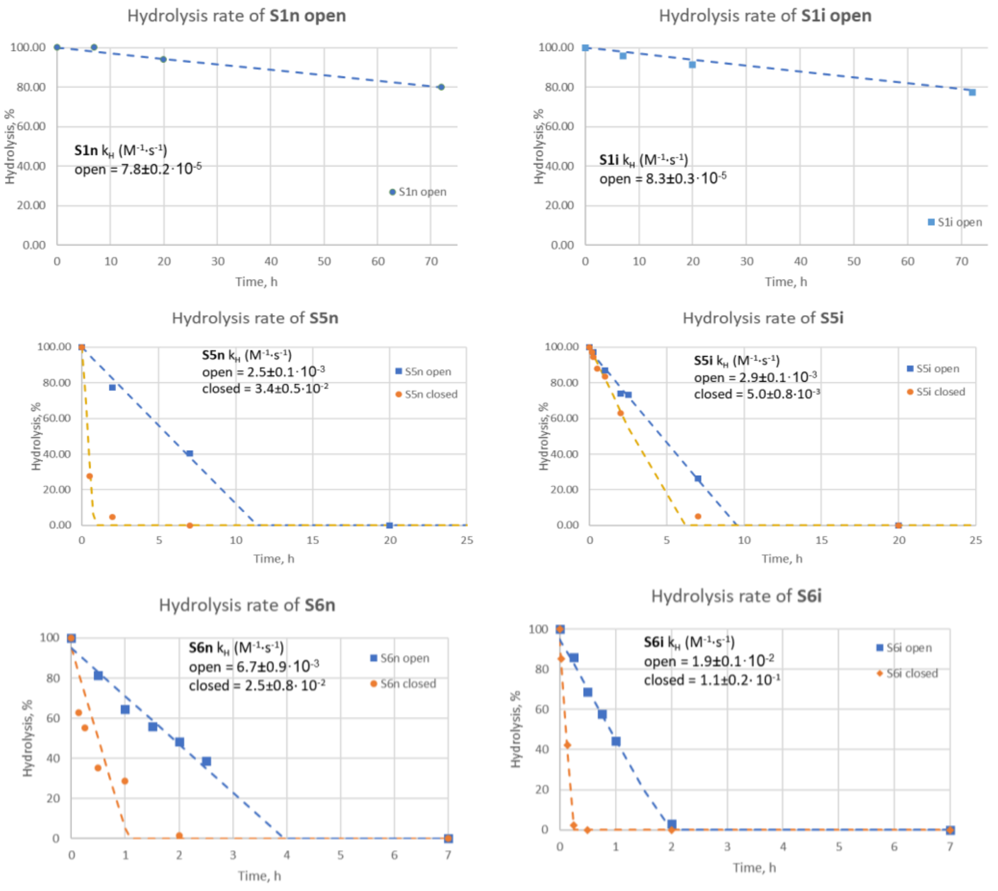

Figure S7 *(continuation)*

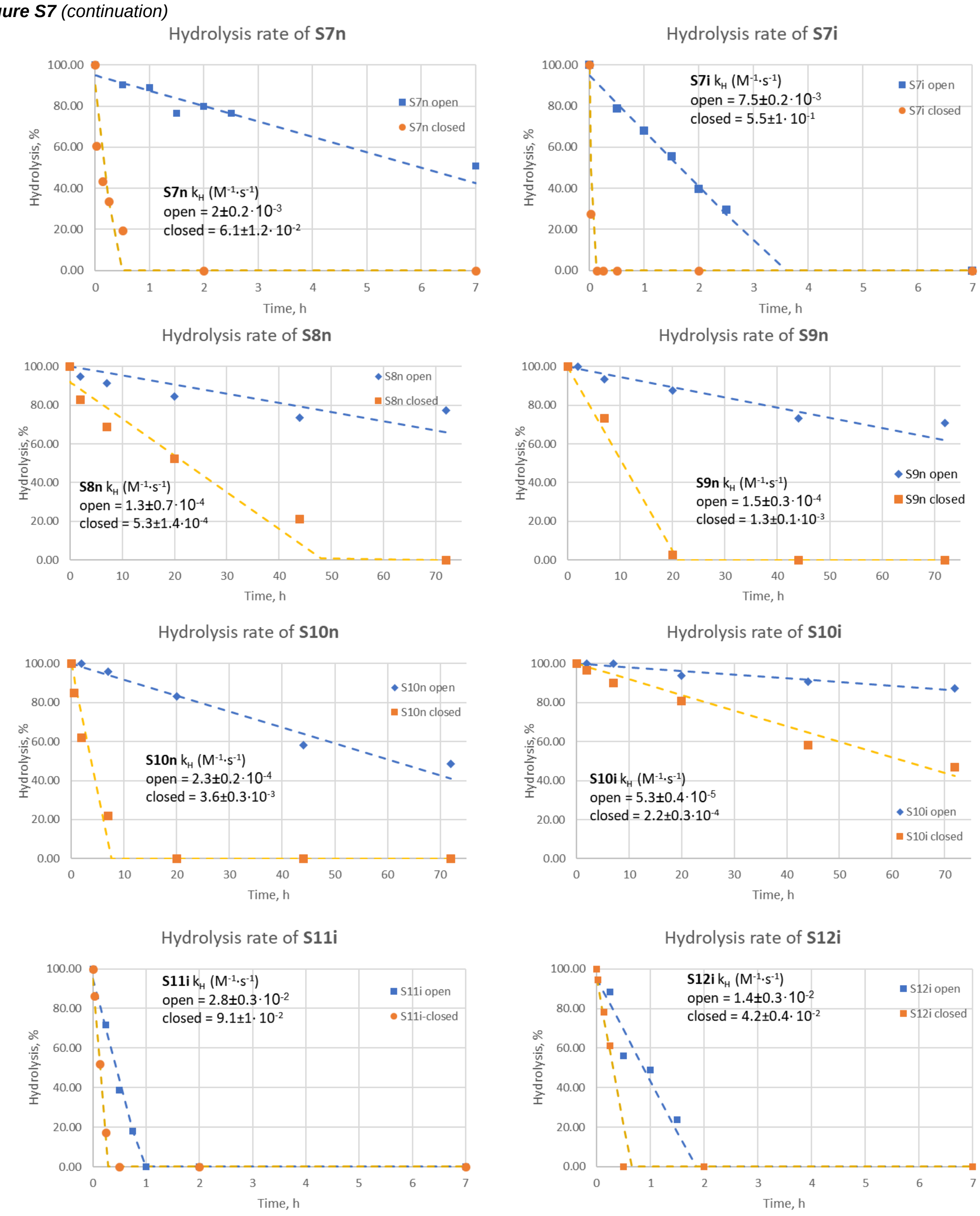

8. Digestion of gelatin-based hydrogels

First, we performed the experiment in vials using constant concentration (10 μM) of either **S10n** ring-open form or **S10n** ring-closed form, to find optimal conditions for the real-time experiment described below. The samples were prepared by dissolving a gelatin powder (food grade) at the concentration of 20 mg/mL in a 9:1 mixture of Milli-Q® water and a Tris-HCl buffer (25 mM $CaCl_2$, 50 mM Tris-HCl, pH 8,2) at 45°C. For gel visualization, all samples were stained with aqueous bromophenol blue (final concentration 25 μg/mL). A double dilution series of a trypsin stock solution ware prepared. They were added as small aliquots to the gelatin-containing glass bottles to obtain the final concentrations 512, 256, 128, 64, 32, and 16 nM, following the peptide addition (aqueous stocks). All operations were done with maximal protection from light exposure. After complete assembly, the samples were cooled to 4°C to solidify the gelatin. Next, the samples were incubated at 20°C for two h in bottom-up bottle positions to monitor the gels' liquefying and subsequently photographed. The result is shown in **Figure S8**.

***Figure S8**. Photocontrolled trypsin digestion of a hydrogel. (A) Without inhibitor, (B) in the presence of ring-open, or (C) ring-closed S10n. The hydrogel was stained by bromophenol blue for better visibility.*

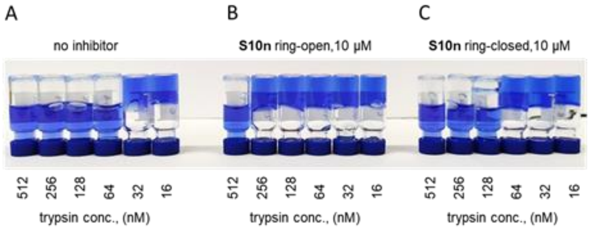

Based on the results of the experiment in vials, we designed a real-time photoswitching experiment with the gelatin-based hydrogel. 20 mL of the bromophenol blue-stained gelatin solution (20 mg/mL) was prepared in a plastic Petri dish. **S10n** ring-closed form was added to a final concentration of 10 μM, followed by trypsin (final concentration 100 nM). The solution was immediately poured to the Petri dish and cooled to 0°C on an ice bath (in the dark). After 10 min, the Petri dish was irradiated with visible light through a mask to convert the photoswitchable inhibitor into active ring-open form. The irradiation was done with LUMATEC light source (Superlite), spectral range 590 nm, power density ~10 mW/cm^2, 3 min exposure time. Afterward, the dish was incubated for two h at 20°C, and the liquid was removed with a filter paper and photographed.

9. Regulation of the anti-bacterial action of peptide (BP100-RW)

The regulation of anti-bacterial action of the antimicrobial peptide BP100-RW was done by its digestion with trypsin protease in a spatiotemporal controlled manner. Gram-negative *Escherichia coli* DSM 498 bacteria strain grown in LB medium was used. The antimicrobial peptide BP100-RW (sequence RRLFRRILRWL-NH_2) was reported to have strong antimicrobial activity with MIC values measured for other *E. coli* strains in MHB medium being 0.2-0.5 μg/ml.[S7]

First, the minimal inhibition concentration in the experimental setup (high initial bacterial inoculation to OD 0.1 in LB medium), was determined. The experiment was performed in single-use polystyrene spectrophotometer cuvettes with covers. 500 μL of LB medium was added to the cuvettes, followed by a calculated volume of a peptide stock solution (0.4 mg/ml) to obtain the target final concentrations of 128, 64, 32, 16, 8, 4, 2, and 1 μg/ml BP100-RW. The calculated volume of bacterial suspension in the exponential growth phase was then added to the cuvettes, including one control without the peptide. Optical density was recorded, and the solutions were incubated on a shaker at 37 °C for 40 min, and optical density was recorded again to assess bacterial culture growth. The results showed that BP100-RW concentration of 32 μg/ml or higher inhibited bacterial growth.

In the second step, the concentration of trypsin required to digest the antimicrobial peptide present at 64 μg/ml (2 x MIC value) in the medium to the concentration safe for bacterial growth (< 32 μg/ml) was determined. 500 μL of LB medium was added to the cuvettes followed by a calculated volume of the peptide stock solution (0.4 mg/ml) to obtain the final concentration of 64 μg/ml. Calculated aliquots of freshly prepared in 1 mM HCl trypsin solution were added to obtain concentrations of 128, 64, 32, 16, 8, 4, 2, 1, 0.5 nM trypsin, and the solutions were incubated at 37 °C for 30 min. Next, the bacteria suspension was added to obtain an OD of 0.1, the solutions were incubated on a shaker at 37 °C for 40 min, and the OD was measured again. The results showed that 1 – 2 nM trypsin is sufficient to deactivate by digestion the action of the antimicrobial peptide.

Finally, the key experiment was designed based on these optimized parameters. Solutions in cuvettes were prepared by adding 500 μL of LB medium and aliquots of antimicrobial peptide (to a final concentration of 64 μg/ml), trypsin (to 3 nM), and **S10n**-closed (to 300 nM); the final volume of the solution was 550 μL. One cuvette was irradiated with visible light for 1 min (570 nm, 143 mW/cm^2, LUMATEC® light source) to convert the peptide inhibitor to the active ring-open form while the other was kept darkened. The solutions were incubated for at 37°C for 30 min, the bacteria culture was added to OD of 0.1 and the bacteria culture growth was checked after 40 and 80 min under incubation on a shaker at 37°C. Control without BP100-RW and with BP100-RW plus trypsin were included. The experiment was conducted 3 times. The results are shown in **Figure S9**.

Figure S9. *Regulation of anti-bacterial action of an antimicrobial peptide BP100-RW by its digestion via the photocontrol of trypsin protease activity.*

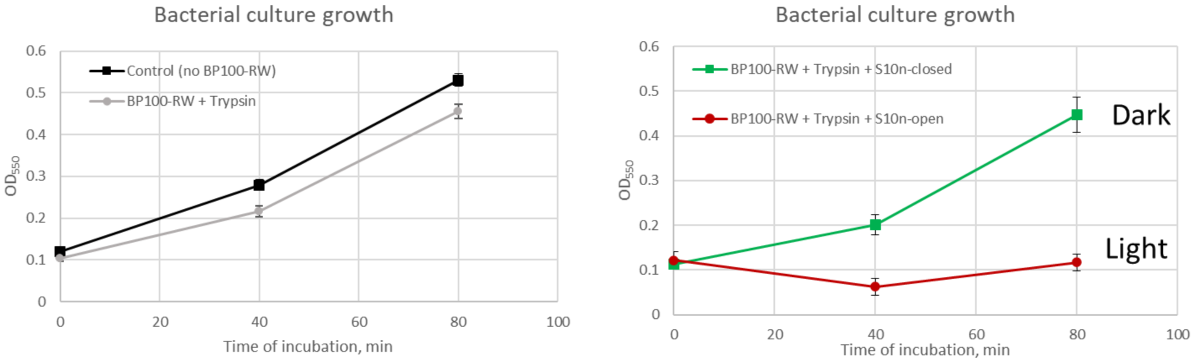

10. Hydrogen/deuterium exchange rates

Hydrogen/deuterium (H/D) exchange rates were determined using MALDI-TOF-MS.[S8] Stock solutions of peptides (200 µM) were prepared in the H_2O/MeCN 2:1 mixture. The experiment was performed in a 10 mM sodium citrate buffer prepared in D_2O, pD 4.5, at 22°C. For the exchange reactions, a 3 µL aliquot of a stock solution was diluted with 70 µL of the citrate buffer in an Eppendorf® reaction tube. At time intervals of 20 s, 40 s, 80 s, 120 s, 300 s, and at the exchange maximum (1 h), each time 3 µL was withdrawn from the incubation mixture and quenched by dilution with 70 µL of 2% formic acid in H_2O/MeCN (2:1), saturated with the MALDI matrix (α-cyano-4-hydroxycinnamic acid). The non-deuterated controls were prepared the same way but using the buffer prepared in H_2O. The quenched solutions were immediately deposited (1 µL) on a stainless-steel target; the droplets were quickly dried in the air stream (using a cold-temperature fan) and analyzed by MALDI-TOF-MS. The spectra were recorded in the positive-ion acquisition mode with the reflectron detector. Matrix and other small ions (below 1000 Th) were suppressed by ion-deflection.

To find the optimal pD for the H/D exchange rates measurements, the peptide **SFTI-1** was incubated at three different pD: 4.5, 5.5, and 6.5 (**Figure S10**). Almost complete H/D exchange was observed already at the first time point (20 s) at pD 5.5 and 6.5. A slower exchange rate was observed at pD 4.5. Therefore, this buffer was chosen for the measurement of the whole peptide set.

The experiment was repeated three times for all peptides. For every time point, the average value of Δ mass was calculated and graphs representing the number of slow-exchanging protons versus exchange time were built. The number of slow-exchanging protons at a given timepoint was calculated as $\Delta\ mass_{(maximum)} - \Delta\ mass_{(timepoint)}$. Measurement for **SFTI-1** were included as controls in each experimental set and provided reproducible results. The data show that peptides have a certain number of slow exchanging protons (from 7 to 2), each with its own $t_{1/2}$, and therefore the curve cannot be fitted to obtain a single $t_{1/2}$ value: plotting of natural logarithm $\ln(H_t)$ as a function of time, where H_t is the number of unexchanged hydrogens at time (t), is not a straight line, indicating that the slow-exchanging hydrogens are heterogenous in terms of their half-times. This is a known and well described phenomenon.[S9c] Therefore, the number of slow-exchanging protons with $t_{1/2}$ longer than a selected value (40 s) was calculated (measured as unexchanged protons at the timepoint of 40 s).[S9c] The obtained H/D exchange versus time curves are shown in **Figure S11**. **Table S6** summarizes the slow-exchanging proton numbers for the studied compounds.

Figure S10 *Incubation time-dependent MALDI spectra for optimization of the H/D exchange experiments for* ***SFTI-1*** *in different D_2O buffers, pD 4.5, 5.5, and 6.5.*

SFTI-1 exchange at pD 4.5

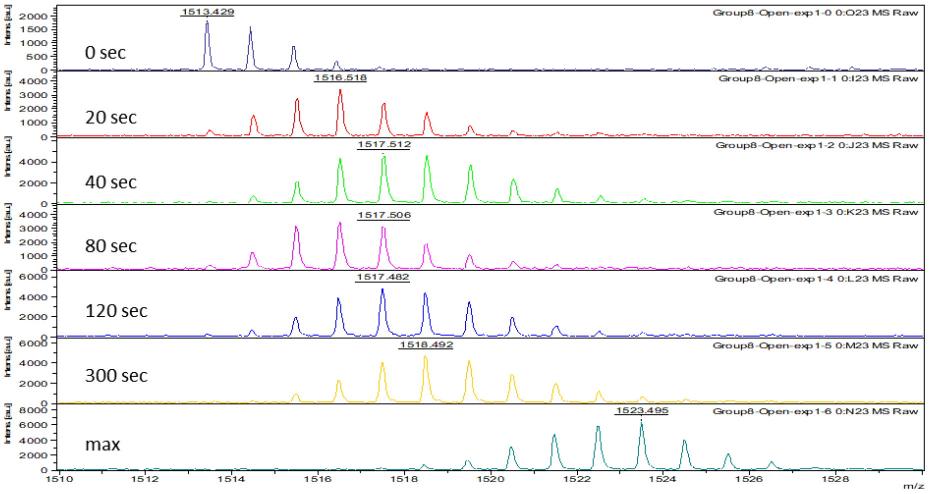

SFTI-1 exchange at pD 5.5

Figure S10 *(continuation)*

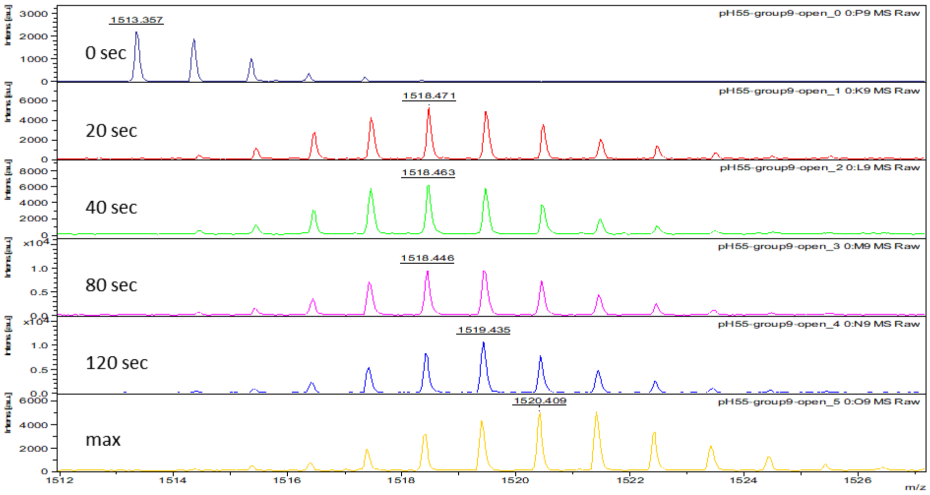

SFTI-1 exchange at pD 6.5

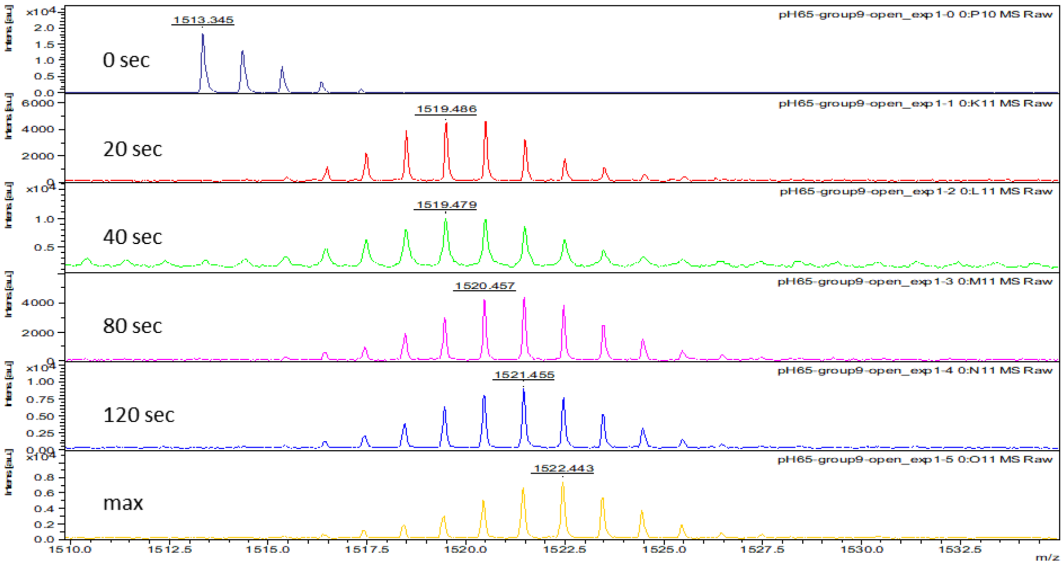

Table S6*. Results of the H/D exchange experiments. The number of slow-exchanging peptide backbone amide protons (measured at the timepoint of 40 s).*

**Peptide**	**Number of slow-exchanging protons**	
	open	*closed*
**SFTI-1**	6.8 ± 0.4	-
**SFTI-1i**	6.8 ± 0.2	-
**S1n**	3.8 ± 0.1	-
**S1i**	4.3 ± 0.3	-
**S5n** open	2.7 ± 0.2	2.9 ± 0.4
**S5i** open	2.9 ± 0.1	2.5 ± 0.1
**S6n** open	3.4 ± 0.1	2.5 ± 0.1
**S6i** open	3.3 ± 0.1	3.3 ± 0.2
**S7n** open	3.4 ± 0.4	2.9 ± 0.5

**S7i** open	3.5 ± 0.2	2.3 ± 0.2
**S8n** open	5.5 ± 0.2	3.5 ± 0.2
**S9n** open	5.7 ± 0.2	4.3 ± 0.1
**S10n** open	6.5 ± 0.1	3.9 ± 0.2
**S10i** open	5.7 ± 0.1	4.1 ± 0.2
**S11i** open	3.7 ± 0.2	3.5 ± 0.1
**S12i** open	4.2 ± 0.2	3.4 ± 0.2

Figure S11. *H/D exchange kinetics. The graphs show the number of not exchanged protons vs time in D_2O citric buffer, pD 4.5 at every incubation time.*

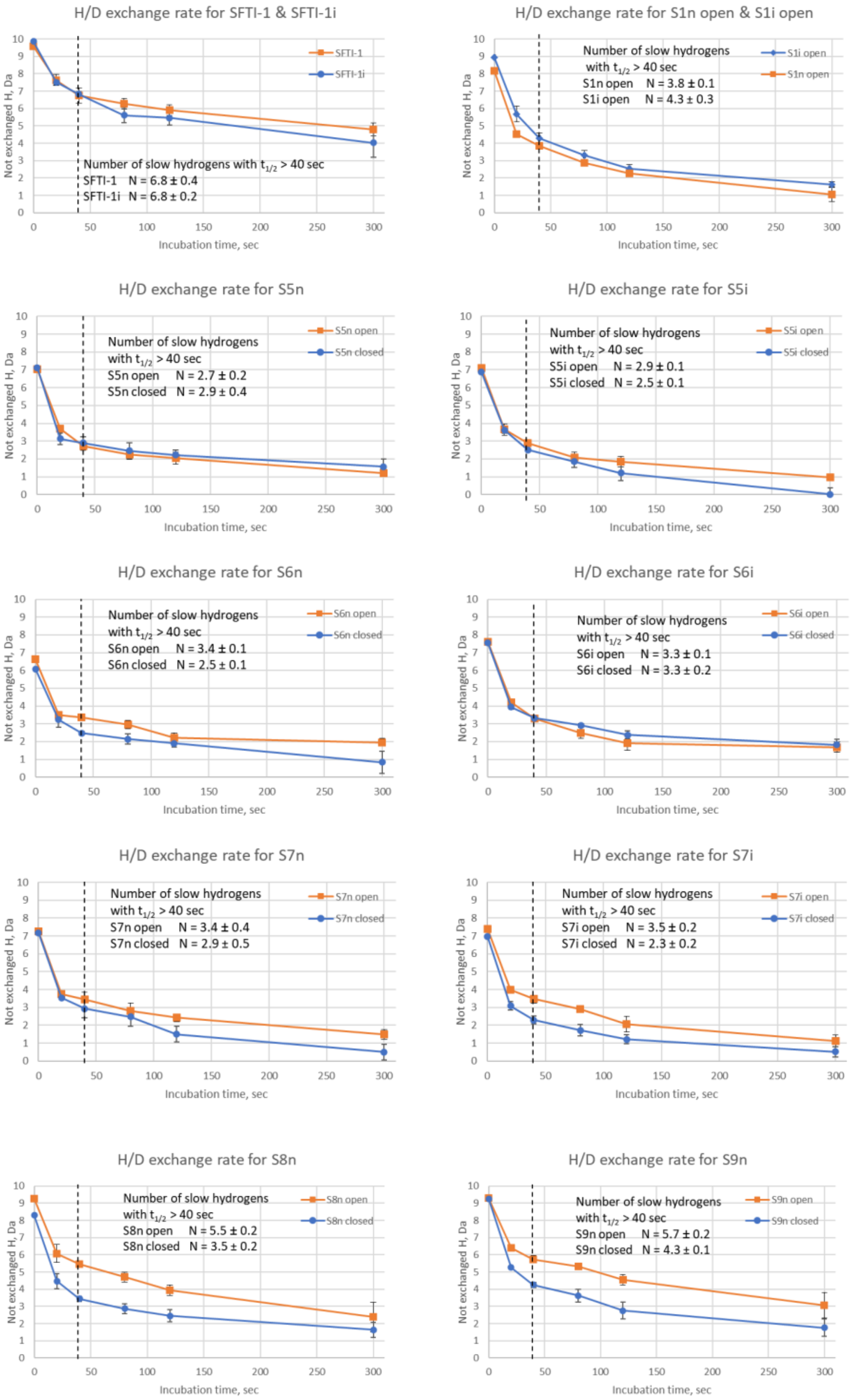

Figure S11 *(continuation)*

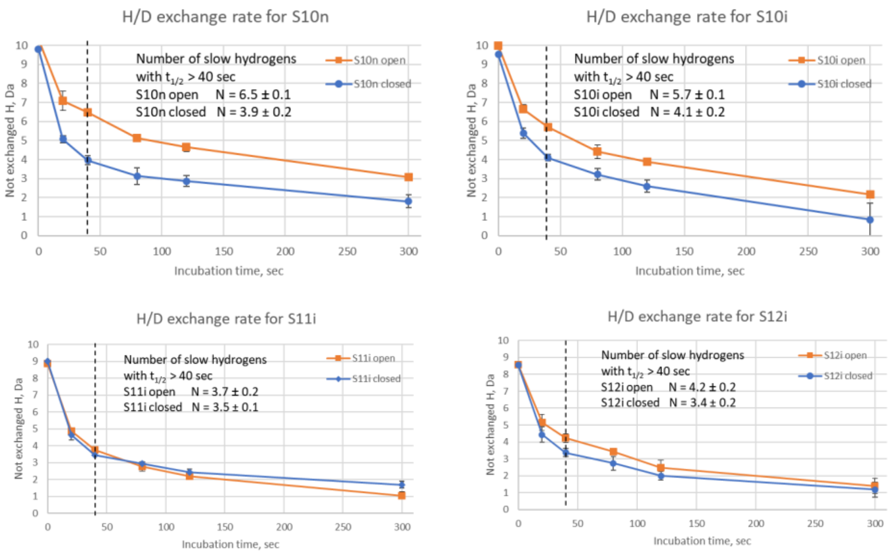

11. HPLC chromatograms

Analytical HPLC chromatograms of **SFTI-1**, **SFTI-1i** and both, ring-open and ring-closed, photoforms of the photoswitchable analogues (whenever available) after purification were acquired, using a 10-60%B gradient (1 min to 51 min) to result in a shallow 1%B per min gradient slope (**Figure S12**).

Figure S12 *Analytical chromatograms for **SFTI-1** and its analogues. Detection at 220 nm.*

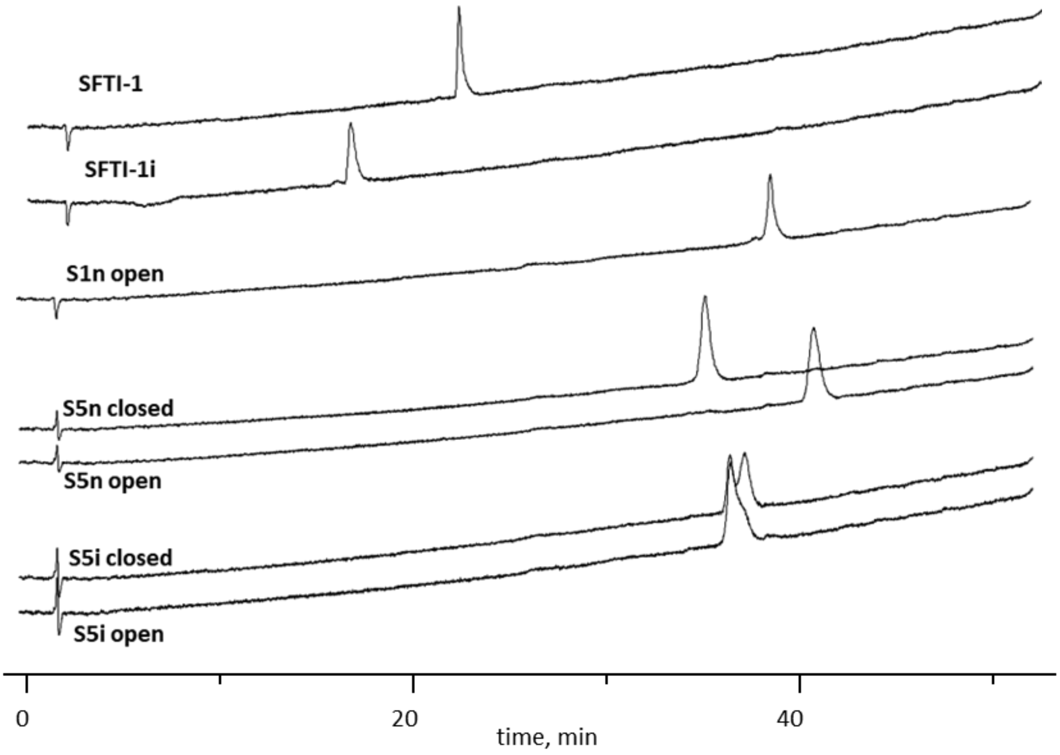

Figure S12 *(continuation)*

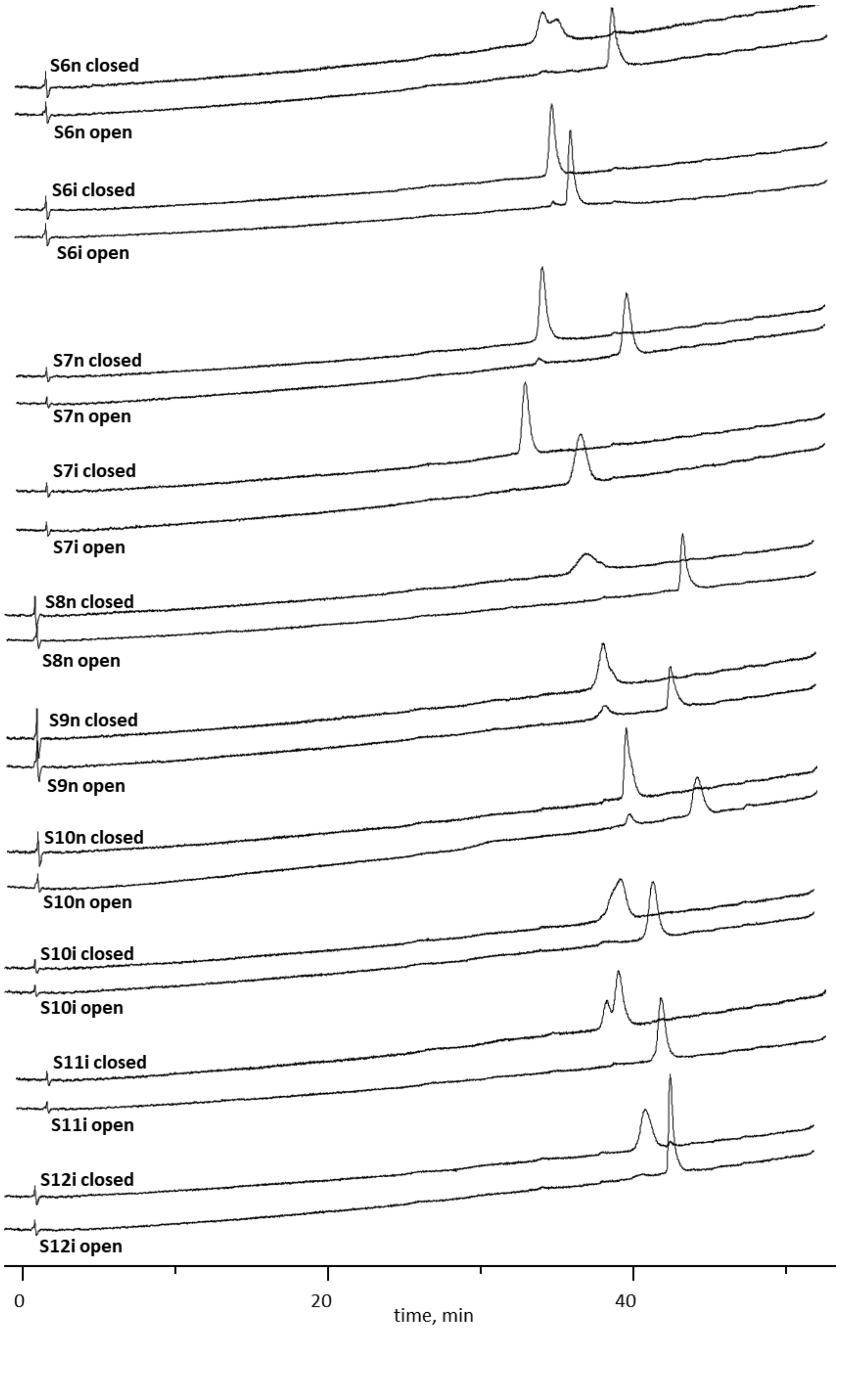

For the peptides, **SFTI-5F** and **S5F** ring-open and ring-closed states, a different gradient (20-70% B in 50 min, 1 min to 51 min) was used due to their more hydrophobic nature (**Figure S13**). Small impurities observed in the chromatograms for the peptide **S5F** were identified by MALDI-TOF-MS as a +2Da, i.e., the compounds with the non-oxidized disulfide bond.

Figure S13 *Analytical chromatograms of the purified chymotrypsin-inhibiting* ***SFTI-5F*** *and its photoswitchable analogue* ***S5F*** *(both photoforms). Detection at 220 nm.*

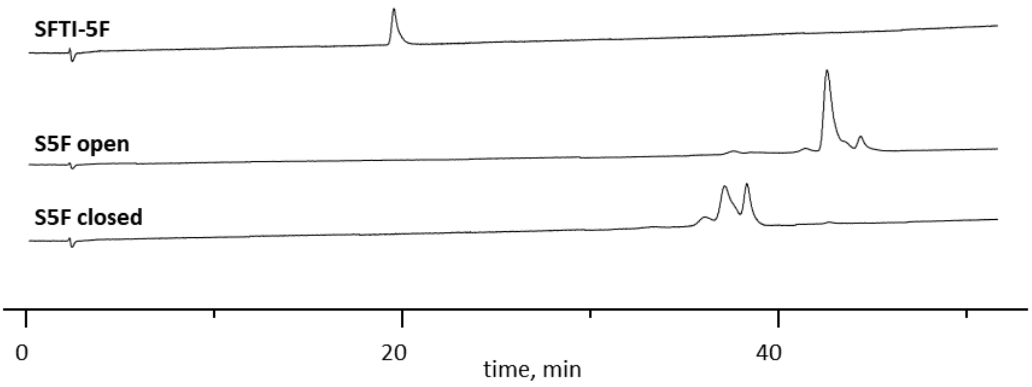

12. MALDI-TOF mass spectra

The identity and purity of the synthesized peptides were confirmed by positive mode MALDI-TOF-MS. The corresponding spectra are shown in **Figure S14** (the calculated masses for the peptides are given in **Table S2**).

Figure S14 *Mass spectra for all studied peptides after purification.*

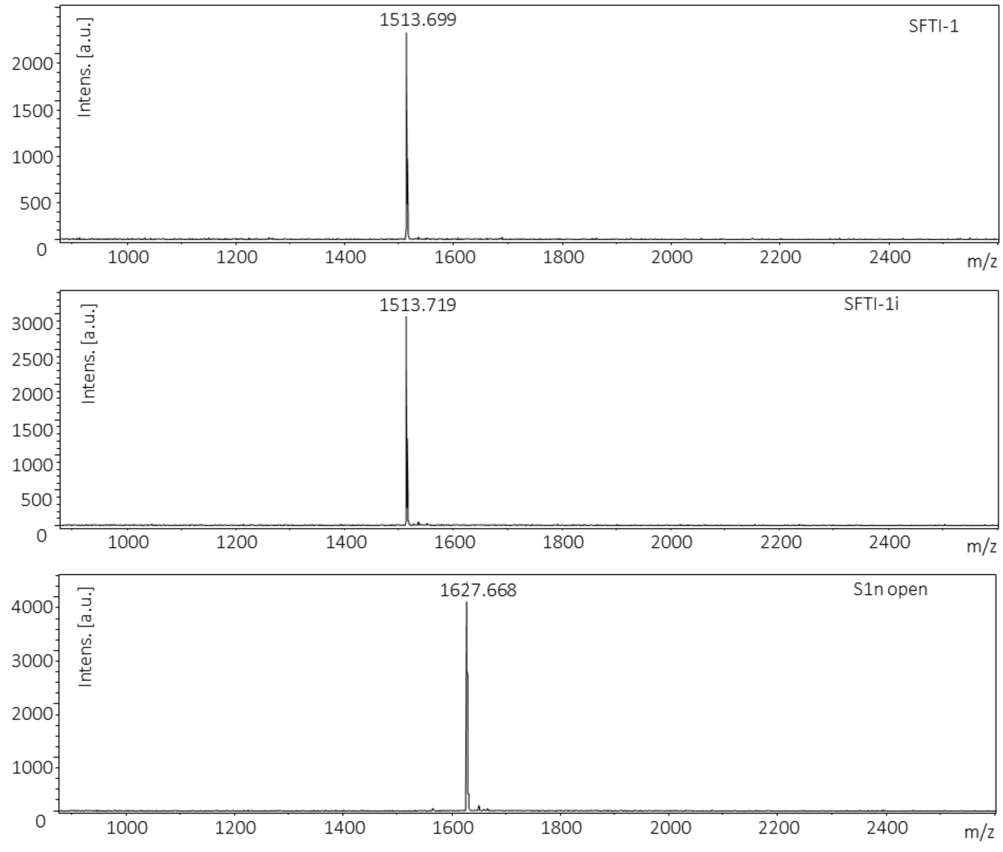

SUPPORTING INFORMATION

Figure S14 *(continuation)*

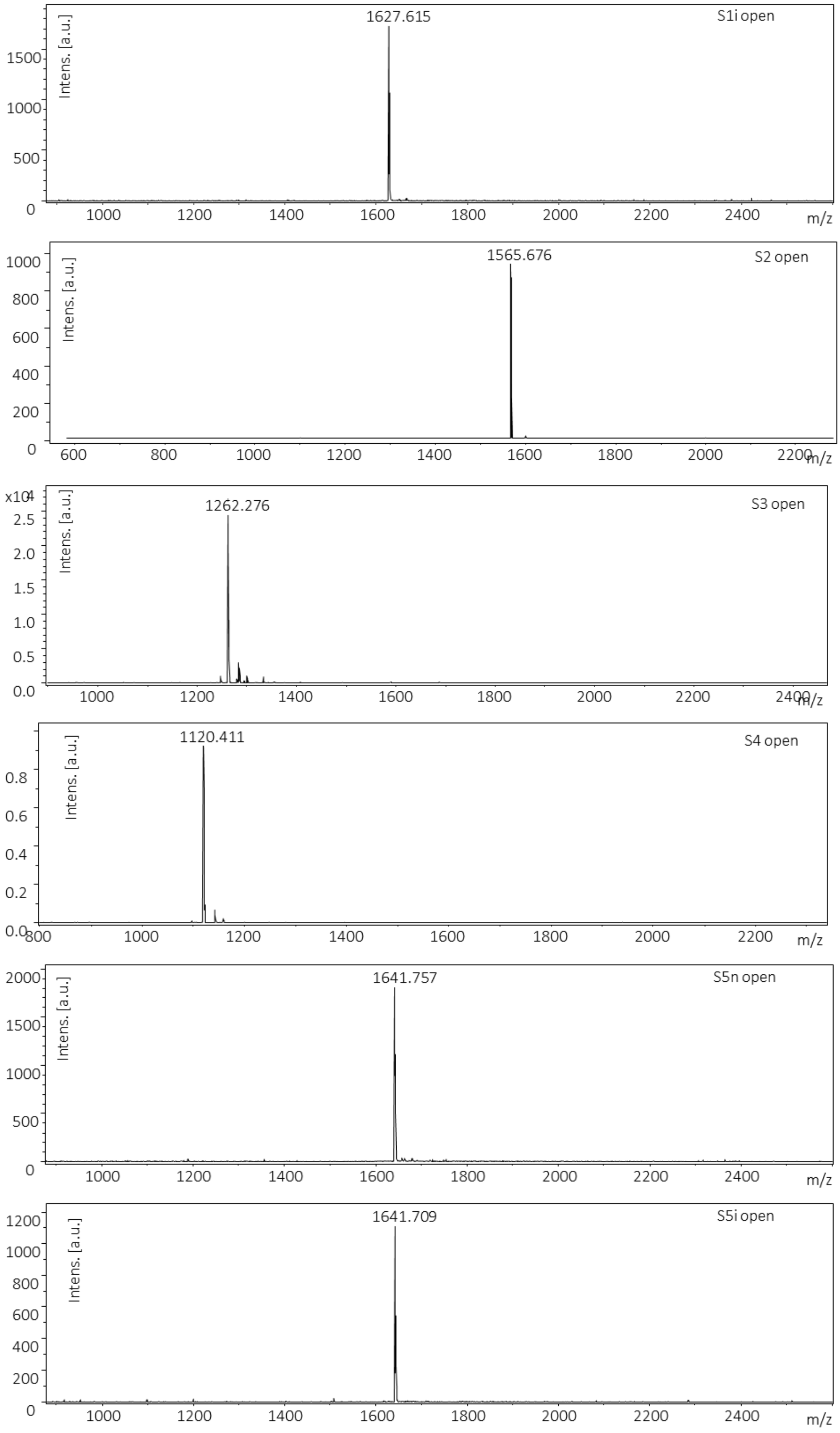

Figure S14 *(continuation)*

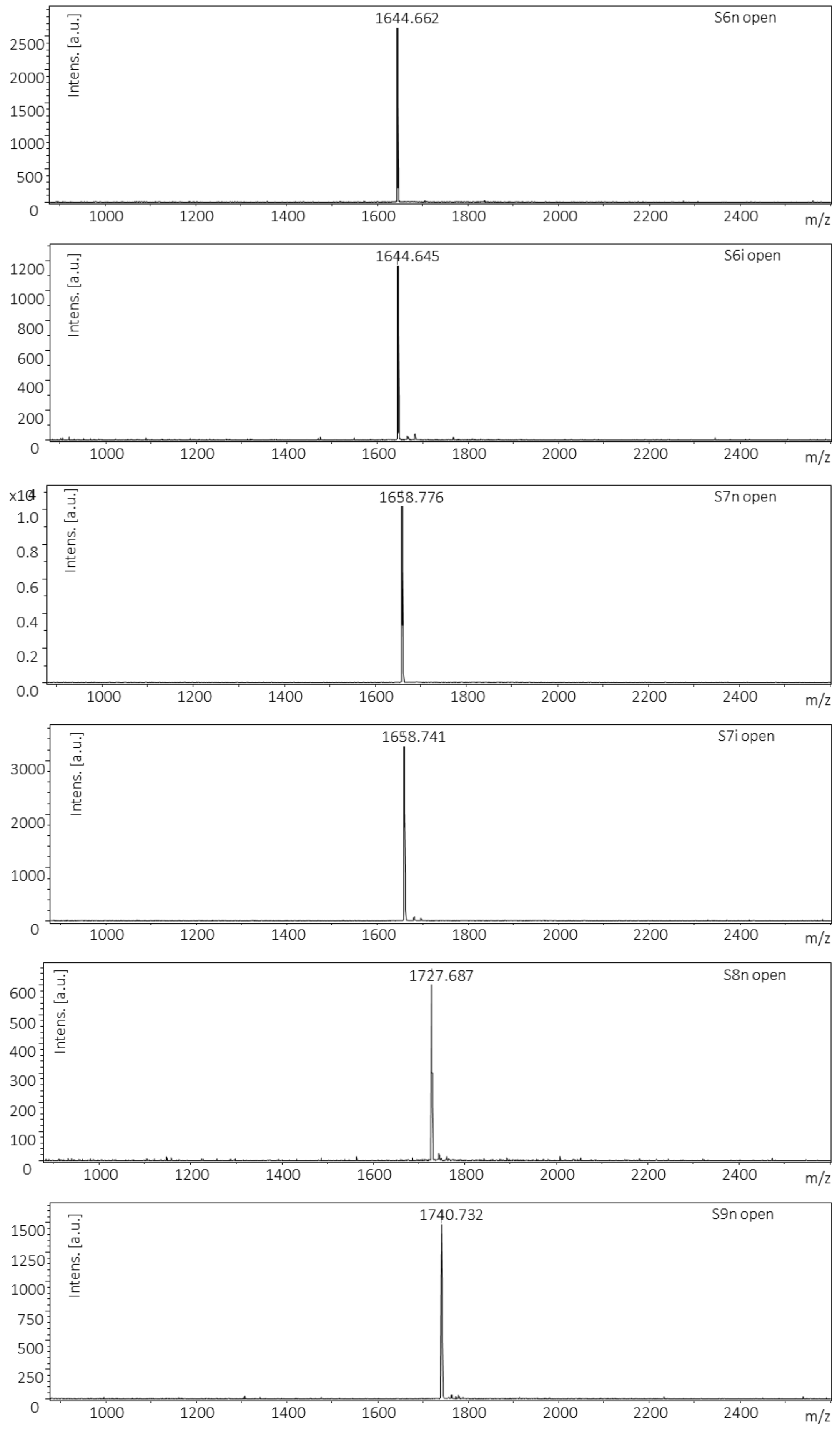

Figure S14 *(continuation)*

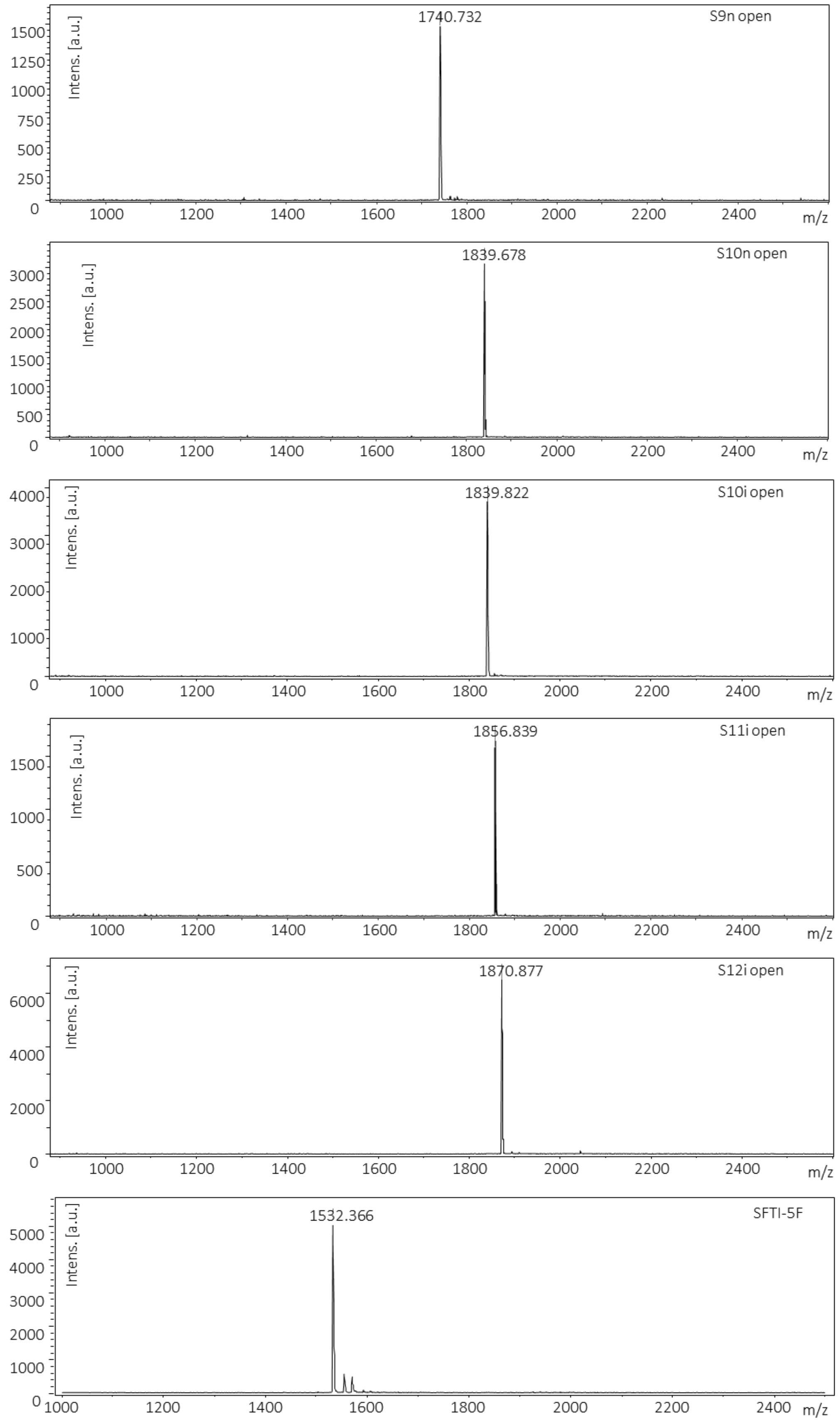

Figure S14 *(continuation)*

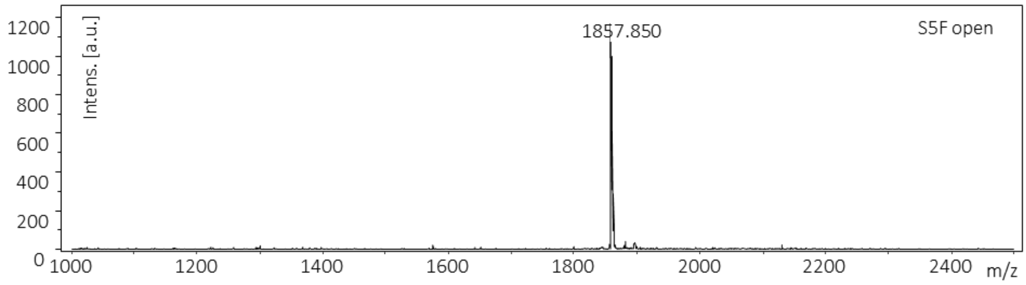

13. References

[S1] O. Babii, S. Afonin, L. V. Garmanchuk, V. V. Nikulina, T. V. Nikolaienko, O. V. Storozhuk, D. V. Shelest, O. I. Dasyukevich, L. I. Ostapchenko, V. Iurchenko, S. Zozulya, A. S. Ulrich, I. V. Komarov, *Angew. Chem., Int. Ed.* **2016**, 55, 5493−5496.

[S2] Fmoc Solid Phase Peptide Synthesis: A Practical Approach; W. Chan, P. White; Eds. Oxford University Press: New York, **2000**.

[S3] P. E., Schneggenburger, B. Worbs, U. Diederichsen, *J. Pept. Sci.* **2010**, 16, 10-14.

[S4] (a) Y. H. Lau, Y. Wu, P. de Andrade, W. R. J. D. Galloway, D. R. Spring, *Nature Protocols*, **2015**, 10, 585-594. (b) V. Hong, S. I. Presolski, C. Ma, M. G. Finn, *Angew. Chem.* **2009**, 121, 10063-10067.

[S5] (a) *Enzymes. A Practical Introduction to Structure, Mechanism, and Data Analysis*; R. A. Copeland; Ed. VCH, Weinheim, **1996**. (b) H. Lineweaver, D. Burk, *J. Am. Chem. Soc.* **1934**, 56, 658-666.

[S6] P. Gonzalez-Tello, F. Camacho, E. Jurado, M. P. Paez, and E. M. Guadix. *Biotechnology and Bioengineering*, **1994**, 44, 523-528.

[S7] M. L. Colgrave, M. J. L. Korsinczky, R. J. Clark, F. Foley, D. Craik, *J. Peptide Sci.*, **2010**, 94, 665-672.

[S8] I. M. Torcato, Y.-H. Huang, H. G. Franquelim, D. Gaspar, D. J. Craik, M.A.R.B. Castanho, S. T. Henriques, *Biochimica et Biophysica Acta*, **2013**, 1828, 944-955.

[S9] (a) T. E. Wales, J. R. Engen, *Mass Spectrom. Rev.* **2006**, 25, 158-170. (b) X. Shi, T. E. Wales, C. Elkin, N. Kawahata, J. R. Engen, D. A. Ann, *Anal. Chem.* **2013**, 85, 11185-11188. (c) Z. Zhang, D. L. Smith. *Protein Sci.*, **1993,** 2, 522-531.
